# Impacts of Basin-Scale Climate Modes on Coastal Sea Level: a Review

**DOI:** 10.1007/s10712-019-09562-8

**Published:** 2019-08-19

**Authors:** Weiqing Han, Detlef Stammer, Philip Thompson, Tal Ezer, Hindu Palanisamy, Xuebin Zhang, Catia M. Domingues, Lei Zhang, Dongliang Yuan

**Affiliations:** 1 Department of Atmospheric and Oceanic Sciences, University of Colorado, Boulder, CO 80309 USA; 2 Center für Erdsystem Wissenschaften und Nachhaltigkeit, Universität Hamburg, 20146 Hamburg, Germany; 3 Department of Oceanography, SOEST, University of Hawai‘i at Mānoa, Honolulu, HI 96822 USA; 4 Center for Coastal Physical Oceanography, Old Dominion University, Norfolk, VA 23508 USA; 5 LEGOS – OMP – CNES, UMR 5566, 31400 Toulouse, France; 6 Centre for Southern Hemisphere Oceans Research (CSHOR), CSIRO Oceans and Atmosphere, Hobart, TAS 7000 Australia; 7 Institute for Marine and Antarctic Studies, University of Tasmania, Hobart, TAS 7004 Australia; 8 ARC Centre of Excellence for Climate Extremes, University of Tasmania, Hobart, TAS 7001 Australia; 9 Antarctic Climate and Ecosystem Cooperative Research Centre, Hobart, TAS 7001 Australia; 10 Key Laboratory of Ocean Circulation and Waves, and Center for Ocean Mega-Science, Institute of Oceanology, Chinese Academy of Sciences, and Qingdao National Laboratory for Marine Science and Technology, Qingdao, China; 11 University of Chinese Academy of Sciences, 266071 Beijing, China

**Keywords:** Coastal sea level change, Sea level variability, Climate variability, Climate modes

## Abstract

Global sea level rise (SLR) associated with a warming climate exerts significant stress on coastal societies and low-lying island regions. The rates of coastal SLR observed in the past few decades, however, have large spatial and temporal differences from the global mean, which to a large part have been attributed to basin-scale climate modes. In this paper, we review our current state of knowledge about climate modes’ impacts on coastal sea level variability from interannual-to-multidecadal timescales. Relevant climate modes, their impacts and associated driving mechanisms through both remote and local processes are elaborated separately for the Pacific, Indian and Atlantic Oceans. This paper also identifies major issues and challenges for future research on climate modes’ impacts on coastal sea level. Understanding the effects of climate modes is essential for skillful near-term predictions and reliable uncertainty quantifications for future projections of coastal SLR.

## Introduction

### Background

Sea level rise (SLR) and extreme events associated with a warming climate are direct threats to human society in low-lying coastal areas and island nations (Wong et al. 2014; Wahl et al. 2018). For adaptation purposes, planners and decision makers require skillful decadal predictions and longer-term projections of SLR along coastlines (e.g., Milne et al. 2009; Church and White 2011; Church et al. 2014; National Research Council (NRC) Report 2012). Producing reliable predictions and future projections of coastal sea level, however, remains challenging due to the complex causes for coastal sea level variations, which differ significantly from the global mean and vary along shore. In addition to non-climate-related local human interferences (e.g., groundwater extraction that causes land subsidence) and geodetic factors, coastal sea level responds to local and remote changes in atmospheric and oceanic circulations. These circulation changes may result from natural internal climate variability, natural external forcing (e.g., solar and volcano) and anthropogenic forcing (e.g., Church et al. 2014; Kopp et al. 2015a, b; Carson et al. 2016).

It is virtually certain (*P* = 0.99) that at least 45% of the global mean SLR over the past century is of anthropogenic origin (Bindoff et al. 2013; Church et al. 2014; Dangendorf et al. 2015; Kopp et al. 2016; Slangen et al. 2016). In contrast, the spatially uneven regional and coastal sea level variability at interannual-to-multidecadal timescales observed since the 1950s has been attributed primarily to internal climate variability, with a large fraction being associated with basin-scale climate modes such as the El Niño and Southern Oscillation (ENSO; e.g., Stammer et al. 2013; Bilbao et al. 2015; Han et al. 2017a). Progress has been made in understanding the impacts of climate modes on basin-wide patterns of sea level variability (Han et al. 2017a). Yet, our understanding of coastal sea level variability caused by climate modes and associated mechanisms is far from complete. In particular, it is unclear to what extent and how remote sea level signals from the open ocean can affect the coasts, as the continental slope can act as a barrier for the communication between the shelf areas and the open ocean (e.g., Brink 1998; Bingham and Hughes 2012; Hughes et al. 2019).

### Purpose of This Review

Given the importance of climate modes in causing open-ocean sea level variability on interannual-to-multidecadal timescales, a thorough understanding of their impacts on coastal sea level is instrumental for improving reliability of decadal predictions and uncertainty quantifications of future SLR projections at coasts. In this paper, we first review the dynamics of communication between coastal and open ocean (Sect. 2); then we summarize the observational evidence for, and our current understanding of, coastal sea level variability from interannual-to-multidecadal timescales induced by climate variability, particularly basin-scale climate modes, in the Pacific, the Indian and the Atlantic Oceans (Sects. 3–5). In Sect. 6, we provide a summary and discuss science issues and future outlook. In this review, unless specified otherwise “decadal-to-multidecadal variability (including multidecadal trend)” is collectively referred to as “decadal” variability (periods ≥ 10 years), and “interannual” refers to periods < 10 years.

## Communication Between the Open Ocean and Coasts: Dynamics

The idea that open-ocean variability can impact coastal sea level has been suggested early on (e.g., Montgomery 1938). Sea level signals in the open ocean and over the shelf communicate through different processes for the eastern and western ocean boundaries. Along the eastern ocean boundaries, sea level anomalies (SLAs) can be significantly affected by signals originated from the equatorial basin through eastward-propagating equatorial Kelvin waves (e.g., Wyrtki 1975) and subsequently poleward-propagating coastally trapped waves after the equatorial Kelvin waves impinge on the coast. If the ocean bottom is flat and coasts are vertical walls, sea level signals along the eastern boundary radiate westward as Rossby waves in regions equatorward of Rossby waves’ critical latitudes, and poleward as coastal Kelvin waves poleward of the critical latitudes. Bottom topography and bottom friction associated with continental shelf and slope, however, can trap part of the incoming equatorial Kelvin waves’ energy to the coast, producing poleward-propagating sea level signals even in regions below the critical latitudes (e.g., Clarke and Vangorder 1994). Compared to meridional coastlines, slanted eastern ocean boundaries affect critical latitudes and somewhat increase the poleward propagation speed, but they do not alter the fundamental nature of coastally trapped sea level signals (e.g., Clarke and Vangorder 1994; Han et al. 2011).

Along the western ocean boundaries, it has been shown that SLAs at particular latitude can be expressed by the sum of contributions from interior SLAs propagating onto the boundary via Rossby waves at and above that latitude, and the western boundary SLAs at higher latitudes via coastally trapped waves (Minobe et al. 2017). This result is based on a linear ocean model that excludes bottom topography and western boundary current. In real coastal oceans, however, continental shelf and slope exist. Under geostrophic approximation and neglecting planetary beta, circulations over the shelf have distinct separations from the large-scale circulations in the open ocean: Currents flow along the isobaths of the continental slope (Brink 1998), setting a “barrier” for cross-isobath flows and thus constraining the remote influence from the open ocean on the coast. The generation of cross-isobath currents must be through ageostrophic processes (e.g., external forcing, nonlinear eddy transport, friction). The long continental slopes in high latitudes also strongly suppresses the effects of open-ocean mesoscale eddies on coasts (e.g., Hughes and Williams 2010; Bingham and Hughes 2012; Hughes et al. 2018, 2019).

By including planetary beta and considering time-dependent large-scale circulations (i.e., greater than Rossby radius), some Rossby waves’ energy is able to cross the “barrier” and arrive at the western boundary, particularly at lower latitude where the topographic effect is weaker due to its dependence on Coriolis parameter (Yang et al. 2013). The degree of the open-ocean impact on coastal sea level depends on bottom friction and on the shapes of the shelf and slope (e.g., width and depth), based on the experiments using a linear model that extends the Minobe et al. (2017) solution by including idealized shelf and slope (Wise et al. 2018). Observational analyses support these theoretical results, showing that offshore sea level is likely to be representative of the coastal signal in areas where the shelf is narrow (e.g., the Florida Straits in the Atlantic), but not likely over the broad shelf of the mid-Atlantic Bight (e.g., Higginson et al. 2015).

At the latitudes of subtropical and subpolar gyres, western boundary currents prevail and they can affect the incoming Rossby waves through advection, and the advection effect is less on the first baroclinic mode Rossby wave compared to higher baroclinic modes (Liu 1999). Observational analyses find that variations of the western boundary currents (e.g., the Gulf Stream and Kuroshio) are significantly correlated with the variability of coastal sea level (e.g., Blaha 1984; Haigh et al. 2011; Ezer et al. 2013; Sasaki et al. 2014). Baroclinic Rossby waves driven by open-ocean wind stress curl propagate westward, affecting the transport of the western boundary currents and therefore the cross-current sea level gradients (i.e., alongshore geostrophy). Given that the cross-shore sea level gradients are weak over the shelf compared to the sea level gradients across the boundary current, SLAs at the inshore side of the boundary current were used to represent coastal SLAs (Hong et al. 2000).

## The Pacific Ocean

In this section, we first provide observational evidence for coastal sea level variability using tide gauge and satellite observations (Sect. 3.1). Since the dynamics of sea level variability along the eastern and western boundaries are different, we review our understanding of the causes for SLAs first along the eastern boundary (Sect. 3.2) and then along the western boundary (Sect. 3.3). For each boundary, we first focus on remote and local processes that induce the observed SLAs and then assess the effects of climate modes. The reviews for the Indian and Atlantic Oceans follow a similar structure.

It is worth noting that satellite altimeters do not observe sea level within 10–20 km of the coast (partly due to land contamination), but they provide a near-global coverage albeit with a relatively short span (~ 26 years since October 1992). In the immediate vicinity of the coast, information about sea level variability relies on tide gauge observations, which provide long records but only at particular locations. Consequently, reconstructed and reanalysis products, which provide near global sea level with relatively long time span (e.g., since 1950s when more in situ data are available), are often used in sea level studies. However, neither the satellite altimetry data over the shelf nor reconstruction or reanalysis products are able to capture the full magnitude of interannual sea level events observed by tide gauges in most coastal areas (e.g., Hamlington et al. 2015).

### Observations

**Fig. 1:**
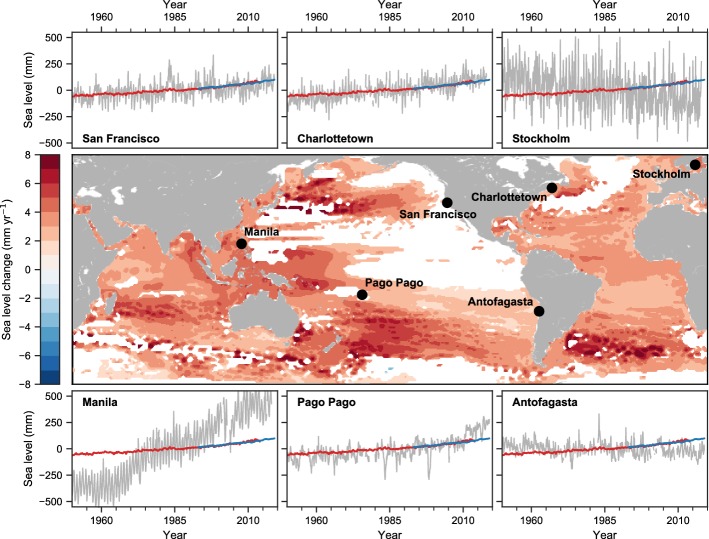
Map of rates of change (trends) in sea surface height (SSH; geocentric sea level) for the period 1993–2018 from satellite altimetry; trend values exceeding 90% significance are shown in color and those below 90% significance are shown in white. Also shown are relative sea level changes (gray lines) from selected tide gauge stations for the period 1950–2018. For comparison, estimates of global mean sea level change from tide gauges (red lines) and satellite altimetry (blue lines) are shown with each tide gauge time series. The relatively large, short-term oscillations in local sea level (gray lines) are due to the natural climate variability, such as the large deviations at Pago Pago of the western Pacific and San Francisco of the eastern Pacific are associated with the El Niño-Southern Oscillation. This is an updated figure of Church et al. (2014)

Satellite altimetry data show unambiguous regional differences in sea level trends since the early 1990s (Church et al. 2014; Fig. 1). Near the eastern boundary of the Pacific, satellite data show no or weak rising trends from 1993 to 2018, and similar situations are also shown in tide gauge data at San Francisco of the North Pacific and Antofagasta of the South Pacific for the same time period (Fig. 1). The multidecadal trend of 1950–2018 at San Francisco tide gauge, however, shows SLR, suggesting that sea level along the Pacific east coast exhibits large-amplitude decadal variability. Indeed, strong interannual and decadal variability has been detected in a number of long, high-quality tide gauge records along the US west coast, and San Francisco holds the longest record since 1854 (e.g., Enfield and Allen 1980; Chelton and Davis 1982; Clarke 1992; Ramp et al. 1997; Meyers et al. 1998; Strub and James 2002; Ponte 2006; Papadopoulos and Tsimplis 2006; Chambers et al. 2012; Breaker and Ruzmaikin 2013; Hamlington et al. 2015). South of the equator, tide gauge records are generally shorter; however, interannual and decadal variability is clearly observed (Fig. 1; Pizarro et al. 2001).

Near the western boundary of the Pacific, satellite data observed marked SLR from 1993 to 2018, with maximum rise occurring in the tropics (Fig. 1). Compared to the eastern Pacific, the western Pacific has complex basin geometry and topography, hosting various marginal seas, broad and narrow shelves and slopes, multitude islands and straits that connect with the Indian Ocean (i.e., the Indonesian Throughflow (ITF); see Sect. 4). These can complicate our understanding of the western boundary sea level variability. The satellite-observed sea level trends in the marginal seas (e.g., the South China Sea, East China Sea and Sea of Japan) and near the ITF straits show the same sign as that of the western Pacific Ocean, indicating that open-ocean signals may exert significant influence on coastal ocean. Consistent with satellite observations, tide gauge data at Manila of the Philippines and Pago Pago in the tropical south Pacific also show SLR from 1993 to 2018, albeit with larger rates. Overlying the multidecadal trends of 1950–2018, sea level at both tide gauges exhibit strong interannual and decadal variations (Fig. 1). Indeed, tide gauges detected coherent multi-scale variability in various subregions along the western boundary of the North and South Pacific (e.g., Senjyu et al. 1999; Church et al. 2006; Sect. 3.3.1).

### Eastern Boundary of the Pacific

#### Remote Versus Local Processes

On interannual timescales, sea level variability along the Pacific eastern boundary is dominated by wind forcing, and the relative contributions of remote versus local wind vary geographically. From the equator to Southern California, interannual SLAs primarily reflect remotely forced, coastally trapped signals of tropical origin (Enfield and Allen 1980; Chelton and Davis 1982). Trade wind anomalies in the equatorial Pacific force eastward-propagating equatorial Kelvin waves; after they impinge upon the eastern boundary, a portion of the energy is reflected back into the ocean interior as Rossby waves, and a portion propagates poleward as coastally trapped waves due to bottom friction and topography, as illustrated by the analytic solutions to a linear ocean model (Clarke and Vangorder 1994) and discussed in Sect. 2. Coherent signatures of these propagating SLAs have been observed as far north as Alaska (Clarke 1992; Ramp et al. 1997; Meyers et al. 1998; Strub and James 2002).

North of San Francisco, however, the impacts of local longshore wind and atmospheric pressure increase with latitude (Enfield and Allen 1980; Chelton and Davis 1982; Hermann et al. 2009). Atmospheric sea level pressure affects sea level via loading, often referred to as inverted barometer (IB) effect (Close 1918; Wunsch and Stammer 1997), which can account for substantial fractions (20–60%) of monthly sea level variance at locations in the Pacific Northwest and Gulf of Alaska regions (Ponte 2006). Longshore winds can produce onshore/offshore Ekman transport, which generates a poleward (equatorward) longshore sea level gradient for southerly (northerly) wind anomalies. The longshore wind and sea level pressure anomalies are, in large part, related to the strength and position of the Aleutian Low (Emery and Hamilton 1985), which is the dominant feature of atmospheric circulation over the Northeast Pacific. Increased strength or eastward displacement of the Aleutian Low raises coastal sea level along the west coast of North America by lowering sea level pressure and increasing the southerly component of wind stress. Along the Canada/Alaska coast, interannual SLAs result from the signals propagating from lower latitudes and those forced by local longshore winds (Qiu 2002).

At decadal time scales, remote tropical forcing—rather than local forcing—is the dominant mechanism. Decadal variations in thermocline depth and sea level of the eastern Pacific as far north as southern California are remotely driven by variability in trade winds of the tropical Pacific (Clarke and Lebedev 1999; Thompson et al. 2014; Merrifield and Thompson 2018). Further north, recent decadal sea level trends at the coast are more closely related to remote equatorial wind forcing with a substantially smaller contribution from local longshore winds (Thompson et al. 2014), even though local forcing is important for interannual SLAs in the higher latitude region; local wind stress curl may also be an important driver (Bromirski et al. 2011), but it is ineffective in accounting for coastal sea level variance in statistical regressions (Chelton and Davis 1982; Thompson et al. 2014). Certainly, wind stress curl is an important driver of open-ocean SLAs (Lagerloef et al. 1995; Fu and Qiu 2002), which propagate westward away from the eastern ocean boundary, leaving the relationship between open-ocean wind stress curl and eastern boundary sea level unclear.

South of the equator, the dynamics of remotely forced SLAs along the eastern boundary largely mirror that of the North Pacific (Enfield and Allen 1980; Pizarro et al. 2001) with interannual SLAs of tropical origin being detected at the southern tip of South America in satellite altimetry (Strub and James 2002; Colas et al. 2008). In contrast to the North Pacific, however, the role of local atmospheric forcing is smaller at higher latitudes, which is reflected by the overall poleward decrease in coastal sea level variance measured by tide gauges (Pizarro et al. 2001). This is at least partially due to the smaller IB effect (< 20%) on monthly mean sea level variance along the South American west coast (Ponte 2006). The role of longshore winds is smaller than in the North Pacific as well, but the effect is not negligible as longshore winds modulate the remotely forced SLAs and account for the seasonal asymmetry between hemispheres in the coastal sea level response to interannual tropical forcing (Strub and James 2002).

#### Effects of Climate Modes

The major modes of climate variability over the Pacific are ENSO (Bjerknes 1969), the Pacific Decadal Oscillation (PDO; Mantua et al. 1997; Zhang et al. 1997), Interdecadal Pacific Oscillation (IPO; Power et al. 1999; Folland et al. 2002) and North Pacific Gyre Oscillation (NPGO; Di Lorenzo et al. 2008). While the PDO is defined as the first empirical orthogonal function (EOF) of monthly sea surface temperature (SST) anomalies in the North Pacific (north of 20°N; with time series of global mean SST removed), the IPO is often regarded as the Pacific-wide manifestation of the PDO (Folland et al. 2002; Trenberth and Jones 2007). On decadal timescales, the indices of PDO, IPO and decadal variability of ENSO are all highly correlated, with correlation coefficients for PDO-IPO and IPO-NINO3.4 (8 year low-passed indices) being both 0.88 during 1900–2008 (Zhang and Church 2012; Han et al. 2014a; Newman et al. 2016). The NPGO is defined as the second EOF of sea surface height anomalies over (180°–110°W, 25°–62°N) of the Northeast Pacific (Di Lorenzo et al. 2008). Since the Southern Annual Mode (SAM) has very weak influence on Indo-Pacific coastal and open-ocean sea level (e.g., Haigh et al. 2011; White et al. 2014; Frankcombe et al. 2015), its effect will not be further discussed in this review.

**Fig. 2:**
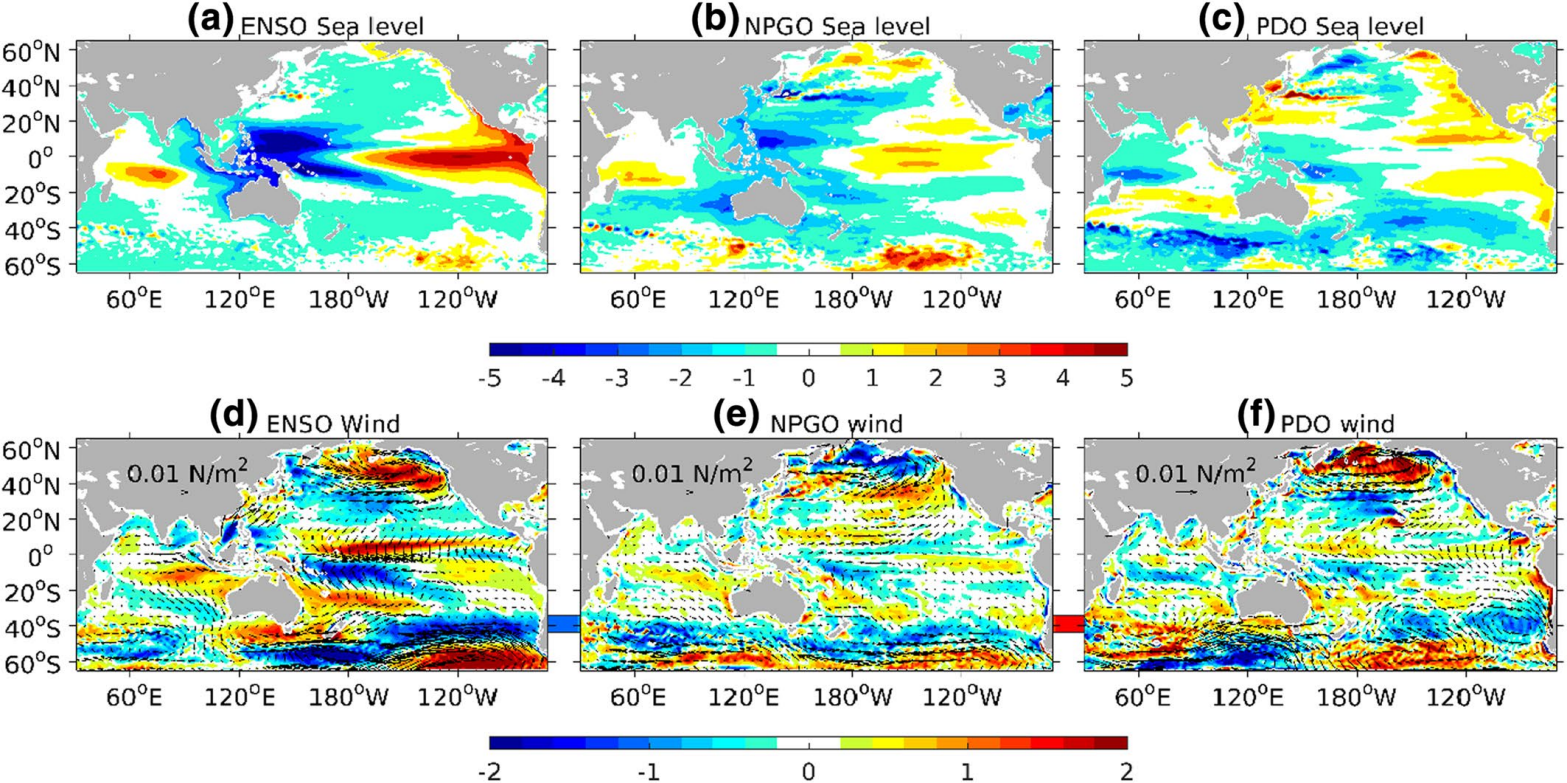
**a** Regression coefficients of sea level (unit: cm) over the Indo-Pacific basin with respect to **a** ENSO, **b** -NPGO and **c** PDO indices based on multiple linear regression analysis of the ECMWF ORAS5 for 1958–2017. **d**–**f**, same as **a**–**c**, but for regression coefficient of wind stress curl (shading; unit: 10–8 N m^−3^) and wind stress (vector). The multiple linear regression is modified from Zhang and Church (2012) by adding the NPGO index, in which sea level anomalies (SLAs) are regressed to temporarily high-passed MEI, low-passed NPGO and PDO indices simultaneously. Note for ease of comparison, the regression coefficients with NPGO index are plotted in panels (**b**) and (**e**). The low-pass filter used here is successive application of a 25- and 37-month running mean to the climate indices, which half power point at a period of 6.2 years. Therefore, the low-pass filtered data contain mainly variability at decadal and longer time scales, while the corresponding high-pass filtered data are derived by removing the low-pass-filtered data from the original data, and contain variability mainly at interannual and shorter time scales (Vimont 2005; Zhang and Church 2012). Regression to the IPO index is similar to that of PDO (Frankcombe et al. 2015)

**Fig. 3:**
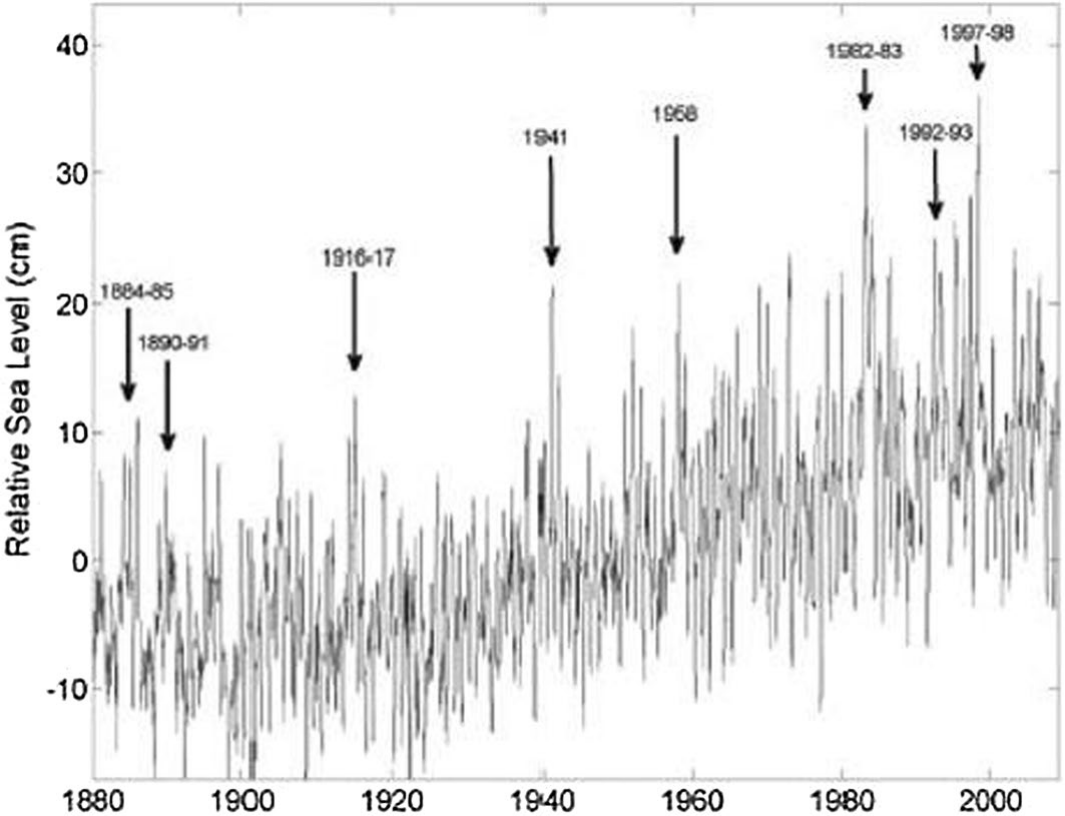
San Francisco tide gauge record showing relative sea level increases during major El Niño events. Tide gage data from the Permanent Service for Mean Sea Level. Figure from NRC report (2012)

Climate modes manifest themselves in basin-wide patterns of sea level variability (Han et al. 2017a). The relative importance of these modes in the basin interior, however, differs from the coastal region along the eastern boundary. Eastern boundary sea level is more closely related to remote and local wind forcing along the equatorial and coastal waveguides, whereas SLAs due to open-ocean forcing propagate westward away from the eastern boundary. Thus, coastal SLAs in the East Pacific are expected to correlate with climate modes that have a strong expression in the tropics and/or atmospheric centers of action that affect longshore winds and sea level pressure. Existing studies demonstrate that interannual SLAs along the East Pacific coasts are highly correlated with ENSO, with El Niño (La Niña) causing large-amplitude coastal sea level rise (fall) (Fig. 2a; e.g., Enfield and Allen 1980; Chelton and Davis 1982; Emery and Hamilton 1985; Clarke and Vangorder 1994; Meyers et al. 1998; Strub and James 2002; Papadopoulos and Tsimplis 2006; Hermann et al. 2009; Thompson et al. 2014; Hamlington et al. 2015). Indeed, large sea level increases during major El Niño events are clearly seen in San Francisco tide gauge data since 1880 (Fig. 3) and are identifiable in Antofagasta tide gauge at the South American west coast since 1950 (Fig. 1).

**Fig. 4:**
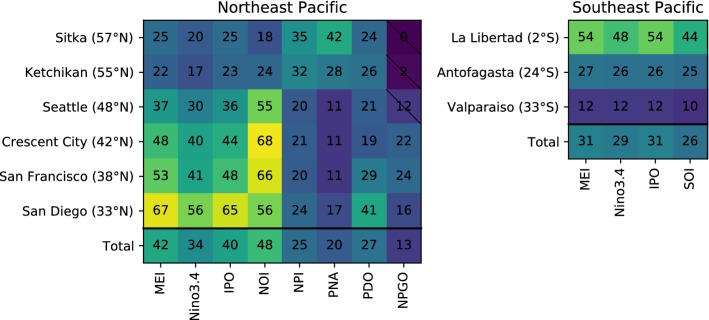
Percentage of interannual and longer variance accounted for by Pacific climate indices in detrended sea level observations from long, high-quality tide gauges in the East Pacific from 1950 to 2017. Colors map to values; large (small) percentages of variance are highlighted in yellow (purple). Values not significant at the 95% level are marked with a diagonal line. “Total” is the ratio between the sum of (variance accounted for by climate mode at each tide gauge record) and sum of (total sea level variance at each record). A low-pass convolution filter was applied to monthly tide gauge data and climate indices to isolate variability with periods longer than 18 months. The analysis was restricted to the period 1950–2017, which is the period spanned by all climate indices considered here and satisfies the requirement of at least 50 years for separating the influence of climate modes from trends in Pacific sea level (Frankcombe et al. 2015). Tide gauge data are from Permanent Service for Mean Sea Level (Holgate et al. 2013), and detrending is performed using the fitted trend from the regression with the index. See Sect. 3.1.3 of the text for the definitions of climate indices

Below, we use the monthly mean sea level from the long and highest-quality tide gauge records to explore the relative importance of climate modes on coastal sea level along the eastern boundary of the North Pacific. In addition, we have also examined the effects of other climate indices (Fig. 4). For each tide gauge and index pair, we perform a least-squares, multiple linear regression of the tide gauge data onto a linear trend and the climate index in question; then the trend is removed before calculating the variance for Fig. 4. We do not perform regressions onto multiple indices simultaneously, because in some cases the indices are highly correlated. Thus, our results reflect the maximum amount of variance that might be attributed to any particular mode or index.

Of particular interest is that the PDO and NPGO are among the least effective climate modes in capturing interannual and decadal sea level variance along the eastern boundary (Fig. 4). To be clear, these modes are dominant drivers of basin-wide patterns of open-ocean sea level variability in the North Pacific (e.g., Han et al. 2017a), but this does not necessarily make them optimal predictors of coastal sea level. The multivariate ENSO index (MEI; Wolter and Timlin 2011) and IPO index (defined here as a SST tri-pole as in Henley et al. 2015) are far more effective as predictors of eastern boundary SLAs at low- to mid-latitudes, presumably because they capture tropical processes that induce the remotely forced, propagating SLAs observed in tide gauge records. In agreement with Frankcombe et al. (2015), the fraction of variance accounted for by the tropical indices decreases with latitude as the influence of local atmospheric forcing becomes more important.

Between San Francisco and Seattle, although ENSO and IPO still explain large fractions of variance, coastal SLAs are most effectively captured by the Northern Oscillation Index (NOI; Schwing et al. 2002), which is defined as the atmospheric sea level pressure difference between Darwin, Australia and the North Pacific High. The NOI is highly correlated with both the MEI and IPO (*r* = − 0.83 in both cases), but the NOI is particularly effective as a predictor of sea level along the US west coast (Fig. 4) due to the inclusion of the North Pacific High as a center of action, which better captures variability in the longshore wind stress there. Such a mechanism has been proposed as an explanation for a large (≈ 80 cm peak to trough) multidecadal fluctuation in San Francisco sea level spanning the late nineteenth and early twentieth centuries prior to the establishment of other gauges in the region (Miller and Douglas 2007). Note that lengthening the filter from 18 months to 7 years to isolate decadal variations results in a substantial increase in the fraction of coastal sea level variance accounted for by the NPGO (not shown), though statistical significance drops below 95% for the three northern most gauges due to the reduction in degrees of freedom caused by the heavier filter. The increase in variance captured by the NPGO is likely due to the fact that at longer time scales it becomes difficult to statistically distinguish between the various modes and indices. Using the seven-year filter, the correlation between the NPGO and NOI increases from 0.44 to 0.58 (both significant at the 99% level), while anticorrelation between the NPGO and IPO increases from − 0.37 to − 0.67 (both at 99% significance).

For high latitudes in the Gulf of Alaska, the most effective predictors of coastal SLAs are the North Pacific Index (NPI; Trenberth and Hurrell 1994) and the Pacific/North American pattern (PNA; Wallace and Gutzler 1981). Both of which are closely related to variability in the Aleutian low with NPI being a direct measure of the Aleutian low intensity and the latter incorporating the Aleutian low as a primary center of action. The NOI, NPI and PNA are significantly correlated with ENSO, IPO, PDO and NPGO, but they are more effective predictors of coastal SLAs, due to the dynamic link between Aleutian low variability and SLAs via the effects on local sea level pressure and longshore winds. This is related to the fact that PDO is an empirical mode and can be caused by both extratropical and tropical processes. This issue is further discussed in Sect. 6.

In the South Pacific, ENSO indices—i.e., MEI, Niño3.4 index and southern oscillation index (SOI)—and IPO, the climate modes that are centered in the tropics, do not capture large fractions of coastal sea level variance outside the tropics (Fig. 4, right panel; Frankcombe et al. 2015). This is partially due to the regional tectonic and seismic activities that cannot be removed from tide gauge data due to the short GPS record as well as the inability of ENSO and IPO indices to capture a greater fraction of the variance. For example, the MEI accounts for less than 30% of the interannual and longer variance at Valparaiso prior to 1977 and more than 50% of the variance during 1993–present. The relationship during the latter period is dominated by the 1997–98 El Niño event, which suggests that the largest tropical events do affect coastal sea level at mid- to high latitudes along the South Pacific eastern boundary and supports previous findings suggesting this connection (e.g., Strub and James 2002).

More recently, sea level in the East Pacific has undergone a shift from near-zero trends during most of the first two decades of satellite altimetry (Bromirski et al. 2011; Thompson et al. 2014) to large positive trends since around 2012 (Hamlington et al. 2016). It is difficult to distinguish the effects of individual climate modes on sea level over the altimetry period alone (e.g., Zhang and Church 2012), as the Pacific indices can be highly correlated over short periods. For example, after applying the 18-month low-pass filter described above, the PDO index is correlated at magnitude 0.65 or greater (and exceeding the 99% significance level) with all of the following indices during 1993–present: MEI, IPO, NOI, SOI, NPI and NPGO. This underscores the importance of process-oriented studies of the relationships between climate indices and coastal sea level, as not all of these indices represent processes with a clear physical link to sea level along the eastern boundary of the basin. Nevertheless, the inflection in decadal sea level trends can be statistically decoupled from canonical ENSO sea level fluctuations during recent decades (Hamlington et al. 2016), which suggests that recent trends may reflect a phase change in basin-scale Pacific climate.

### Western Boundary of the Pacific

#### Remote Versus Local Processes

**Fig. 5:**
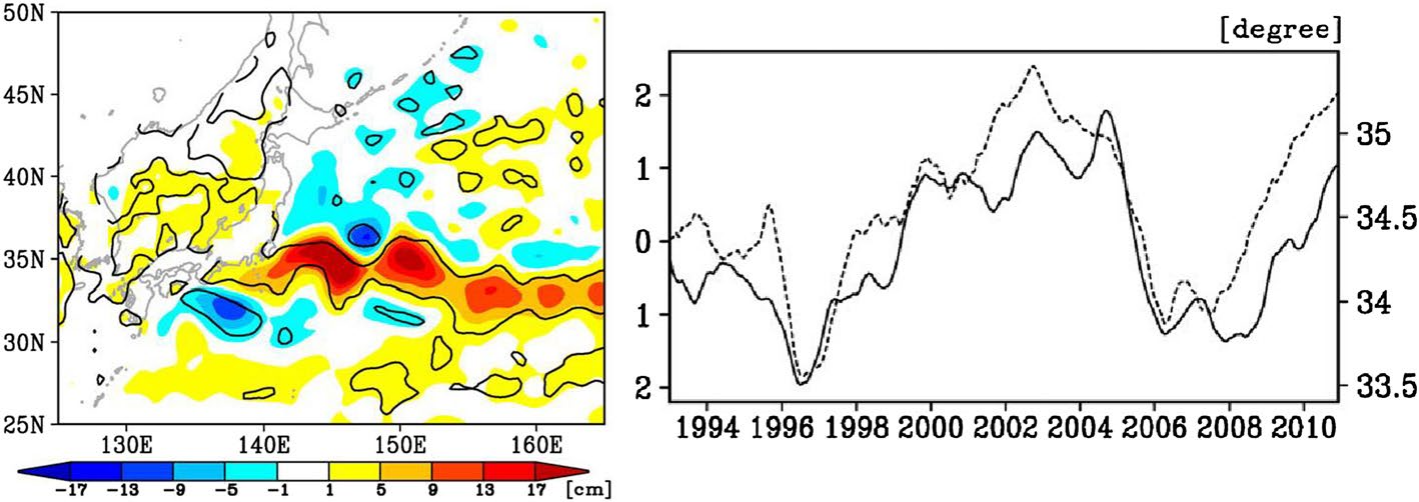
(Left) Regression coefficient map of satellite SLAs onto the normalized time series of the 1st SVD mode (between satellite and tide gauge) from satellite data during 1993–2010, which is the solid curve on the right panel; the line contour indicates the regions where the corresponding correlations are significant at 95% confidence level. (Right) Normalized time series of the 1st SVD mode of satellite SLA (solid line; left axis) and the latitude (dashed line; right axis) of the Kuroshio Extension jet averaged for 140°E–165°E. From Sasaki et al. (2014)

In the subpolar western North Pacific, interannual SLAs along the east coast of Kuril islands are determined by the wind-driven, westward-propagating baroclinic Rossby waves (Qiu 2002). In the Sea of Japan, ocean general circulation model (OGCM) experiments suggest that the tide-gauge observed bidecadal variability of sea level around Japanese coastlines results from the westward propagation of Rossby waves forced by winds in the central and eastern North Pacific basin (Yasuda and Sakurai 2006). Prominent decadal variability of sea level was found along the Kuroshio Extension jet, due to the westward-propagating Rossby waves from the east Pacific (Fig. 5) causing north–south shifts of the jet and thus inducing SLAs around the Japanese coasts, as observed by both tide gauge and satellite data from 1993 to 2010 (Sasaki et al. 2014). The SLAs are large along the southeast coast of Japan due to the direct influence of the jet-trapped Rossby waves, and along the west coast of Japan likely due to coastally trapped waves excited by the incoming Rossby waves.

Along the China east coast, sea level variations are strongly influenced by coastally trapped waves (Hsueh and Pang 1989; Hsueh and Yuan 1997). Over the continental shelf of the East China Sea, local wind stress curl is suggested to be the cause for decadal SLAs (Moon and Song 2017). In the South China Sea, Rossby waves from the western tropical Pacific impinge on the east coast of the Philippines, where their associated SLAs propagate clockwise as coastally trapped waves, causing interannual-to-decadal SLAs around the Philippine coasts (e.g., Liu et al. 2011; Zhuang et al. 2013; Cheng et al. 2016). The effect of local longshore wind is important for SLAs along the west coasts of Borneo and Palawan islands (Cheng et al. 2016).

In the western tropical Pacific, interannual and decadal SLAs around the multitude of islands and ITF straits are primarily attributed to the variability of the easterly trade winds. On interannual timescales, variations in zonal wind in the equatorial Pacific cause SLAs along the Arafura/Australia shelf break via equatorial Rossby waves, which excite coastally trapped waves off the western tip of New Guinea, with portion of the energy entering the Indian Ocean through the Indonesian Archipelago (e.g., Clarke 1991; Wijffels and Meyers 2004; also see Sect. 4). Theoretical studies suggest that SLA signals in the western equatorial Pacific and on the western boundaries of the major landmasses should be in phase and have similar amplitudes based on the island rule (Clarke 1991; Godfrey 1996). Recent observations suggest that nonlinear interactions between the Rossby waves and the Mindanao Current result in significant elevation of sea level in the northern Indonesian seas, which forces significant ITF transport into the Indian Ocean (Yuan et al. 2018).

On decadal timescales, rapid SLR in the western tropical Pacific during recent decades, which opposes the sea level fall in the eastern tropical Pacific basin, has been detected by satellite, tide gauge, reconstructed and reanalysis data (Fig. 1; e.g., Church et al. 2006; Merrifield 2011; Zhang and Church 2012). Both observational analyses and ocean modeling experiments show that the steady intensification of the trade wind is the primary cause for the rapid SLR since the early 1990s (Merrifield 2011; McGregor et al. 2012; Merrifield and Maltrud 2011; Qiu and Chen 2012; Meyssignac et al. 2012; Nidheesh et al. 2013; Han et al. 2014a; Hamlington et al. 2014; Palanisamy et al. 2015). Furthermore, intensified amplitudes of SLAs at 8–20 year periods since the late 1980s were observed and attributed to the intensified decadal variability of easterly trade and its associated off-equatorial wind stress curl (Han et al. 2014a). Interannual and decadal fluctuations of the easterly trade, which are linked to climate modes and inter-basin interactions (see Sect. 3.3.2), cause the west–east seesaw of decadal SLAs (e.g., Carton et al. 2005; Bindoff and Willebrand 2007; Kohl et al. 2007; Lombard et al. 2009; Timmermann et al. 2010; McGregor et al. 2012; England et al. 2014; Han et al. 2014a; Merrifield and Thompson 2018). Local land subsidence, however, may also be an important factor in the amplification of relative SLR (e.g., Ballu et al. 2011; Becker et al. 2012).

In the western South Pacific, the leading EOF pattern from 16 tide gauge records around the Australian coasts explains 69% of the total sea level variance, with larger amplitudes occurring at the northern and western Australian coasts of the Indian Ocean, from where SLAs propagate counterclockwise around the coasts as coastally trapped waves with magnitude decreasing with distance (White et al. 2014). Direct influence from the western equatorial Pacific on SLAs along the Australian east coast is weak, because there is no direct oceanic pathway. Instead, westward-propagating Rossby waves from the subtropical Pacific interior directly impact coastal sea level and the East Australian Current (EAC; Holbrook et al. 2011), with the Indian Ocean (via coastally trapped waves) and Southern Ocean having much weaker influence (White et al. 2014). Interannual-to-decadal variations of EAC transport and SLA measured at the Fort Denison tide gauge in Sydney Harbor are significantly correlated; Rossby waves generated by winds in the Tasman Sea explain the large variances of EAC transport and coastal sea level, and remotely forced Rossby waves from the South Pacific interior account for the multidecadal sea level trend along the New South Wales coast (Holbrook et al. 2011). Observational analysis and OGCM experiments showed that decadal variations of wind stress curl in the South Pacific interior excite baroclinic Rossby waves, causing decadal SLAs along the east coast of New Zealand; subsequently, Rossby waves emanated from New Zealand propagate into the Tasman Sea, causing decadal SLA along Australian southeast coast and the coasts of Tasmania (Sasaki et al. 2008).

#### Effects of Climate Modes

The trade wind anomalies that cause the sea level east–west seesaw and interannual variability in the western tropical Pacific result primarily from ENSO (Fig. 2; e.g., Nerem et al. 1999; Landerer et al. 2008; Zhang and Church 2012; Wu et al. 2017). Sea level is low (high) in the western tropical Pacific during El Niño (La Niña), and its variability exhibits pronounced seasonality and north–south asymmetry. During El Niño, low sea level appears north of the equator (e.g., Guam and Marshall islands) from July to December and south of the equator (e.g., American Samoa islands) from January to June, due to the seasonal evolution of westerly wind anomalies in the western and central equatorial basin (Chowdhury et al. 2007). On decadal timescales, the IPO phase transitions are linked to SLAs in the western tropical Pacific, such as the rapid SLR since the early 1990s (e.g., Merrifield et al. 2012; Meyssignac et al. 2012; Zhang and Church 2012; Hamlington et al. 2013, 2014; Moon et al. 2013; Han et al. 2014a; Palanisamy et al. 2015; Lyu et al. 2017). The intensified wind and SLAs during recent decades, however, cannot be fully explained by the IPO (Han et al. 2014a); decadal variability of SST over the tropical Indian and Atlantic Oceans also had significant contributions (Luo et al. 2012; Han et al. 2014a; McGregor et al. 2014).

In the western North Pacific, ENSO influence on coastal SLAs weakens with latitude, as shown by the analysis of 27 tide gauges along the China Coastline from 1968 to 2016 and satellite altimetry (Wang et al. 2018). From 1993 to 2015, ENSO-SLA correlations are the largest in the South China Sea (> 0.6 in most areas; also see Cheng et al. 2015), weaker in the East China Sea (> 0.4 in most areas) and weakest in the Bohai and Yellow Seas (< 0.4), with all correlation coefficients being statistically significant at the 95% confidence level. Significant correlations between PDO index and interannual SLAs (up to 10 cm) have also been found in the northwest Pacific including the East China Sea (Papadopoulos and Tsimplis 2006; Han and Huang 2008). On decadal timescales, SLAs are significantly correlated with the PDO in the East China Sea (Han and Huang 2008) and South China Sea in recent decades, due to Rossby waves forced by PDO-associated wind stress curl propagating to the South China Sea through the Philippine Archipelago (Cheng et al. 2016). Moon and Song (2017), however, found that decadal SLAs in the East China Sea in the past 50 years were highly correlated with the NPGO rather than PDO (also see Merrifield 2011), although the PDO can explain the multidecadal trend reversal since the mid-1980s. Indeed, either NPGO or PDO can dominate coastal SLAs, depending on locations and periods (Fig. 2).

In the western South Pacific, variations of the wind stress curl that drive interannual-to-decadal Rossby waves and coastal SLAs along the east coasts of Australia and New Zealand and around the coasts of Tasman Sea are associated with ENSO (e.g., Sasaki et al. 2008; Haigh et al. 2011; Holbrook et al. 2011; White et al. 2014). As discussed above, due to the lack of direct oceanic pathway the impacts of ENSO and IPO on sea level along the Australian east coast are not as strong as those along the north and west coasts (Fig. 2; e.g., Zhang and Church 2012; Frankcombe et al. 2015; Wu et al. 2017).

Wu et al. (2017) examined the interannual and decadal (6-year low-passed) SLAs as well as trend associated with ENSO and PDO over 1993–2011 and found that the SLAs and trend were well explained by the steric component in the open ocean, with a major contribution from the thermosteric component while the halosteric component only played a minor role in the western equatorial Pacific region. Over the shallow shelves of the western Pacific boundary, however, mass component plays a significant role (their Fig. 4).

## The Indian Ocean

### Observations

**Fig. 6:**
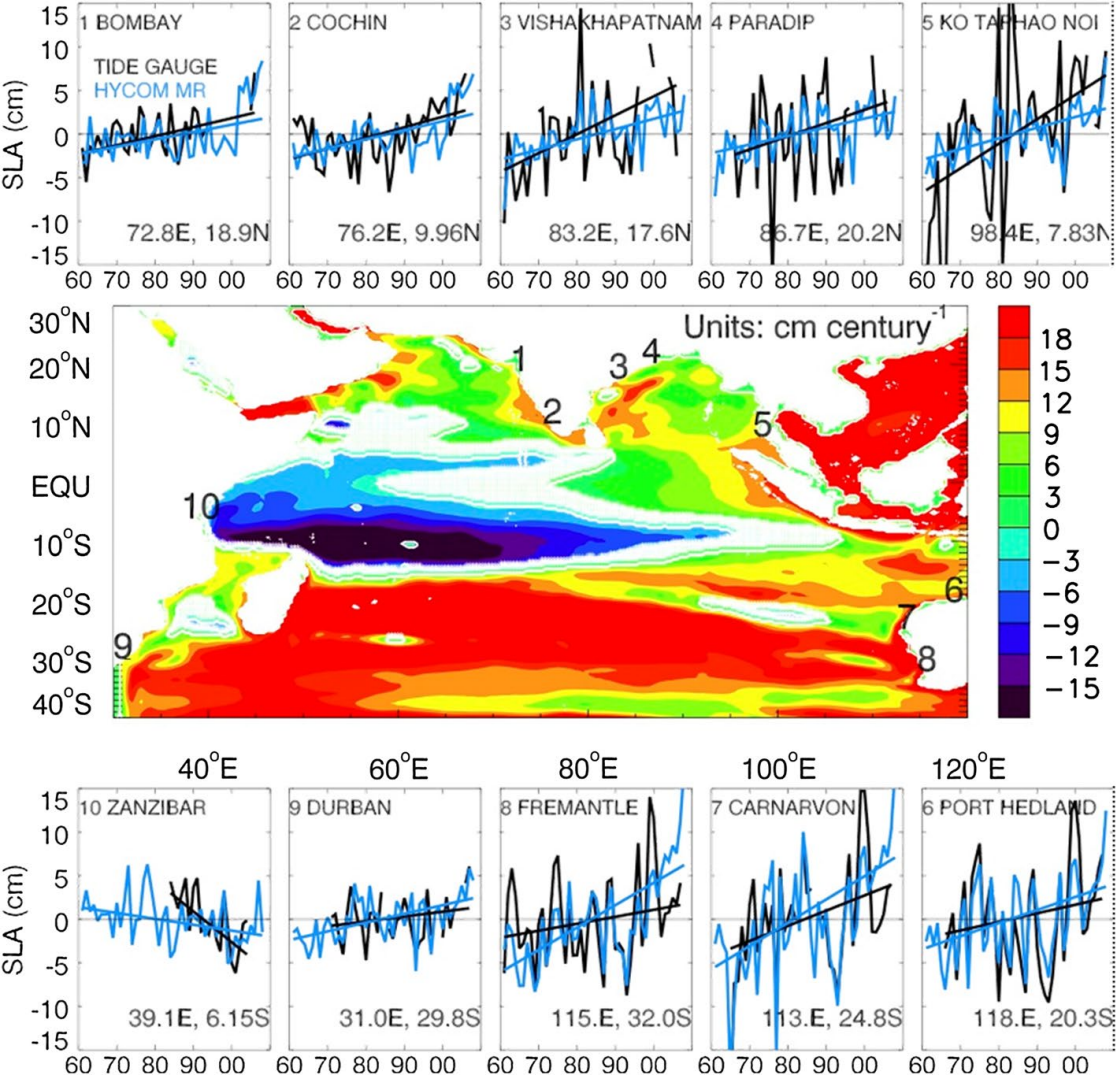
Tide gauge observed and HYCOM simulated annual mean sea level anomalies (SLA) and their Kendall Theil trends during 1961–2008. The 10 tide gauge stations with records longer than 30 years (20 years for Zanzibar) are shown. All trends exceed 95% significance except for stations 6 and 9 tide gauge data. Middle color panel shows Kendall Theil trend of HYCOM simulated SLA for 1961–2008. Light blue/green regions are below and the rest above 95% significance. Tide gauge locations are marked by 1–10. Customized from Han et al. (2010)

Tide gauge observations show evident multidecadal trends and interannual-to-interdecadal sea level variability around the coastal and island regions of the Indian Ocean (e.g., Shankar and Shetye 1999; Unnikrishnan and Shankar 2007; Han et al. 2010; Frankcombe et al. 2015; Unnikrishnan et al. 2015; Parekh et al. 2017; black lines of Fig. 6). Centennial trends of SLR are also detected at Mumbai, the west coast of India (Shankar and Shetye 1999) and Fremantle, the west coast of Australia (Feng et al. 2004), the only two tide gauges within the Indian Ocean with record lengths longer than 100 years (Bradshaw et al. 2015). The rates of SLR and amplitudes of sea level variability, however, differ considerably from region to region. In particular, a falling trend was detected in a ~ 20-year tide gauge record in recent decades at Zanzibar, the east coast of Tanzania and the western boundary of the Indian Ocean, while rising trends were observed at all other tide gauges since the 1960s/1970s.

Overlying the multidecadal trend, coastal sea level exhibits strong interannual and decadal variations (Fig. 6), with apparently larger amplitudes along the coasts of the east basin (stations 3–8 around the Bay of Bengal and Australian coasts) compared to that of the west basin (stations 1–2 of the Indian west coast and 9–10 of the South African coast). These results agree with the variance map of Shankar et al. (2010), showing the SLA minima in the central equatorial basin and eastern Arabian Sea, but large variance along the boundaries of the East Indian Ocean. Indeed, tide gauge observations detected coherent interannual SLAs along the 8000 km coastline from Java to Mumbai (Clarke and Liu 1994).

**Fig. 7:**
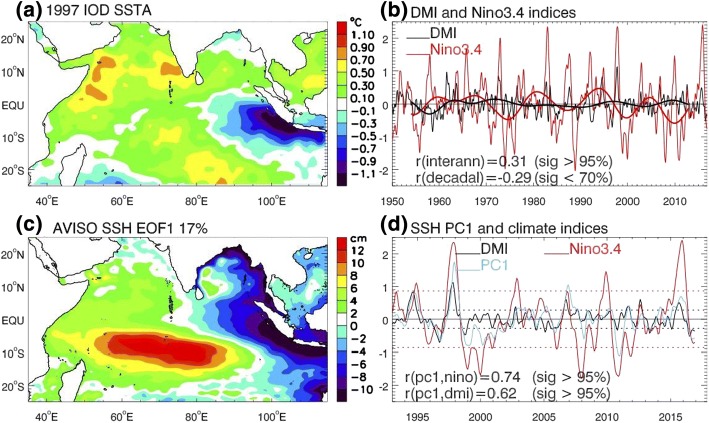
**a** September, October and November (SON) mean SSTA for the 1997 positive IOD-El Nino co-occurrence, based on detrended and demeaned HadISST data from 1950 to 2012; **b** 5-month running mean Dipole Mode Index (DMI; thin black curve) based on monthly SSTA, defined as SSTA difference between western pole (50°E–70°E, 10°S–10°N average) and eastern pole (90°E–110°E, 10°S–0°N average); thin red curve shows 5-month running mean Nino3.4 index; corresponding thick curves are their 8-year low-passed indices; **c** EOF1 of monthly AVISO SSHA (linear trend and monthly climatology removed); **d** SSHA PC1 (blue), DMI and Nino3.4 indices. Horizontal dashed lines show the ± 1STD of DMI (black) and Nino3.4 (red) index

Satellite altimetry since the early 1990s (Fig. 7c) and various reanalysis and reconstructed sea level products since the 1960s show strong interannual-to-decadal SLAs over the Seychelles islands and Chagos Archipelago regions of the tropical South Indian Ocean where the thermocline is shallow (McCreary et al. 1993; Murtugudde et al. 1999), referred to as the Seychelles–Chagos thermocline ridge (SCTR; e.g., Hermes and Reason 2008; Yokoi et al. 2008). Large spread, however, exists among different reanalysis and reconstructed products, suggesting large uncertainties in detecting sea level variability using these datasets (Nidheesh et al. 2017). Decadal variations are evident, although the magnitudes are weaker than that of interannual (e.g., Shankar and Shetye 1999; Feng et al. 2004, 2010, 2011; Holgate and Woodworth 2004: Nidheesh et al. 2013; Figs. 6 and 7d). Indeed, decadal reversals of basin-wide sea level trend patterns have been observed in satellite data (e.g., Lee and McPhaden 2008; Thompson et al. 2016; Srinivasu et al. 2017). The leading EOF of satellite SLAs for 1993–2016 agrees with the tide gauge results, showing larger amplitudes around the boundaries of the east basin and over SCTR, compared to the weaker magnitude in the central equatorial basin and east Arabian Sea (Fig. 7c, d).

### Eastern and Northern Indian Ocean Boundaries and the Seychelles-Chagos Thermocline Ridge (SCTR) Region

Unique to the Indian Ocean, its northern boundary is located in the tropics and its eastern boundary is separated by the ITF, which connects the Indian and Pacific Oceans, the only low-latitude connection between ocean basins in the world. As a result, sea level signals generated in the equatorial basin can propagate southward to Sumatra and Java coasts (e.g., Wijffels and Meyers 2004) and northward into the Bay of Bengal until the west coast of India along equatorial and coastal waveguides (Clarke and Liu 1994), and those signals generated in the equatorial Pacific can exert strong influence on the west coast of Australia (i.e., southeast coast of the Indian Ocean).

#### Remote Versus Local Processes

The multidecadal sea level trends observed by tide gauges around the coasts are associated with a basin-wide spatial pattern, with sea level fall in the tropical southwest basin (including Zanzibar) accompanying SLR elsewhere (including all other tide gauge stations; map of Fig. 6). Similar patterns have been shown in various reconstructed and reanalysis sea level products (e.g., Church et al. 2004; Hamlington et al. 2011; Dunne et al. 2012; Han et al. 2018) and in ocean model simulations (e.g., Timmermann et al. 2010; see Han et al. 2014b and 2017a for reviews). The changing surface winds over the Indian Ocean are the major driver for this distinct pattern (Han et al. 2010; Timmermann et al. 2010; Schwarzkopf and Böning 2011), although the ITF has a significant contribution to the SCTR sea level fall in an OGCM experiment (Schwarzkopf and Böning 2011; Han et al. 2014b). Natural internal variability dominates external forcing in causing the spatially uneven distribution of sea level trends (Fig. 2 of Han et al. 2018). Over the SCTR region, the falling rate of sea level obtains its maximum, and internal variability (external forcing) contributes ~ 81% (19% ± 2.4%) after global mean SLR removed, based on the reanalysis data and large ensemble climate model simulations.

On interannual timescales, the observed interannual SLAs (up to 10–25 cm) in the Bay of Bengal result mainly from surface wind forcing, with influence from buoyancy flux being weak (Han and Webster 2002). Equatorial wind is the main driver of SLAs along the eastern and northern Bay boundaries, by exciting equatorial Kelvin waves and subsequently coastally trapped waves. In the western Bay (i.e., east coasts of India and Sri Lanka), wind within the Bay has a comparable effect as equatorial wind especially during the summer monsoon season, because longshore winds around the Bay generate coastally trapped waves and wind stress curl in the Bay interior cause westward-propagating Rossby waves. Near the Australian west coast, remote forcing by wind from the equatorial Pacific is much more important than local forcing for determining interannual SLAs (e.g., Feng et al. 2003; Wijffels and Meyers 2004; Trenary and Han 2012; White et al. 2014; Deepa et al. 2018), even though composite analyses showed that during some seasons (e.g., May), forcing over the Indian Ocean has a larger effect (Fig. 11 of Trenary and Han 2012). Recent theoretical and modeling studies showed that the sloping ocean bottom plays a crucial role in trapping the Leeuwin current to the coast, even though some energy radiates offshore via Rossby waves (Furue et al. 2013; Benthuysen et al. 2014).

**Fig. 8:**
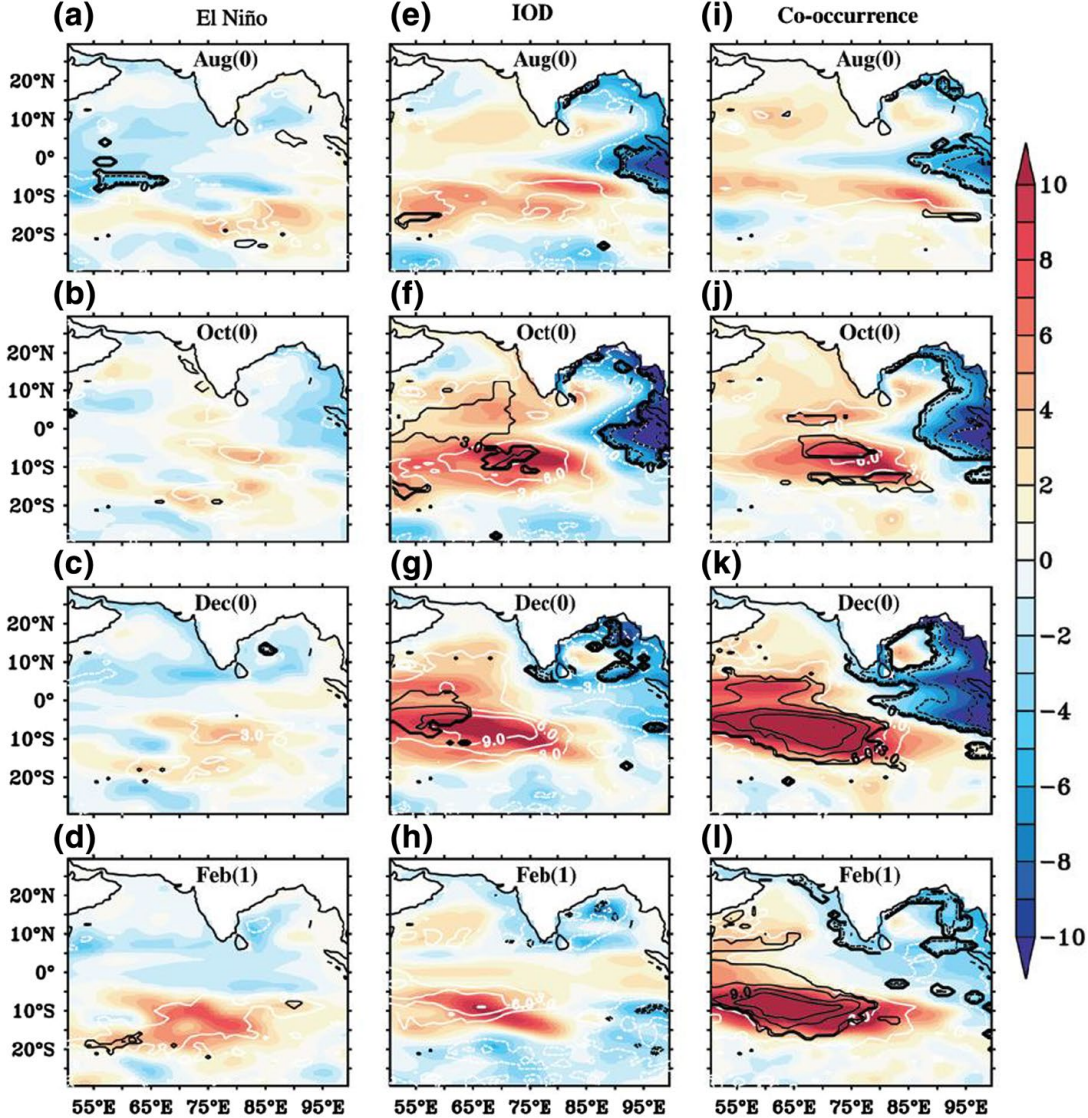
Spatial SLA composite (cm) during El Niño years of 1965, 1976, 1986, 1987 and 2002 (**a**–**d**); pure positive IOD years of 1961, 1967, 1977 and 1994 (e–h); and co-occurrence years of 1963, 1972, 1982, 1997 and 2006 (**i**–**l**) for the months August, October, December and February based on ORAS4 reanalysis (color shaded) and OGCM experiment (white contours) from 1959 to 2008. Black contours are OGCM results significant at 90% confidence level. Figure from Deepa et al. (2018)

**Fig. 9:**
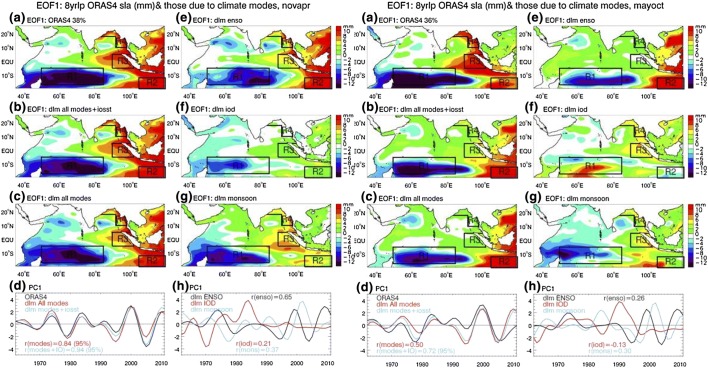
A (left two columns): The leading Empirical Orthogonal Function (EOF1) of the 8-year low-passed northern winter (November–April) mean SLA over the tropical Indian Ocean for the period of 1962–2011 from (**a**) ORAS4 data; (**b**) Bayesian DLM simulation from the sum of all modes and IOSST (ENSO + IOD + monsoon + IOSST); and (**c**) DLM simulation from all modes (ENSO + IOD + monsoon); (**d**) the principal component of EOF1 (PC1) for ORAS4 (black), all modes + IOSST (blue), and all modes (red); (**e**)–(**g**) are the same as (**c**) except for the DLM simulation from ENSO, IOD and monsoon, respectively; (**h**) Same as (d) except for the PC1 of ENSO (black), IOD (red) and monsoon (blue). The correlation coefficients shown in (**d**) and (**h**) are with the observed ORAS4 PC1 (the black curve of panel (d)). Boxes mark the large-amplitude SLA areas. Figure 9B (right two columns): Same as Fig. 9A but for northern summer season of May–October. From Han et al. (2018)

In the central equatorial basin (e.g., the Maldives) and east Arabian Sea (e.g., Indian west coast), interannual SLAs attain their minima (Figs. 7c, 8 and 9), due to the destructive interference between the direct wind-forced signals and reflected Rossby waves from the eastern ocean boundary (Shankar et al. 2010). Over the interior South Indian Ocean, interannual SLAs obtain the maximum in the SCTR region, and they are primarily driven by westward-propagating Rossby waves forced by wind stress curl east of the SCTR (e.g., Tozuka et al. 2010; Trenary and Han 2012), with local wind forcing also playing a non-negligible role (Tozuka et al. 2010).

On decadal timescales, surface wind over the Indian Ocean is suggested to be the dominant forcing for SLAs in most regions (Nidheesh et al. 2013; Trenary and Han 2013; Li and Han 2015), and equatorial wind variability is crucial for driving decadal SLAs in the Bay of Bengal (Nidheesh et al. 2013). Along the west coast of Australia, decadal SLAs are strongly influenced by remote forcing from the trade wind anomalies in the equatorial Pacific (e.g., Feng et al. 2004, 2010; Deepa et al. 2019), although local forcing also has comparable contributions (Trenary and Han 2013). While surface wind is an important driver, changes in salinity due to regional precipitation may also play a role in some regions. For instance, the century-long-tide gauge at Mumbai observed interdecadal sea level variations, which mimic the variability of monsoon rainfall over the Indian subcontinent, suggesting the influence of halosteric effects on coastal sea level (Shankar and Shetye 1999). While thermosteric sea level dominates the observed SLAs in most regions, halosteric sea level also has apparent contributions in some areas, such as the north and west coasts of Australia and the coasts of Bay of Bengal and India (e.g., Fukumori and Wang 2013; Nidheesh et al. 2013; Llovel and Lee 2015; Wu et al. 2017).

#### Effects of Climate Modes

**Fig. 10:**
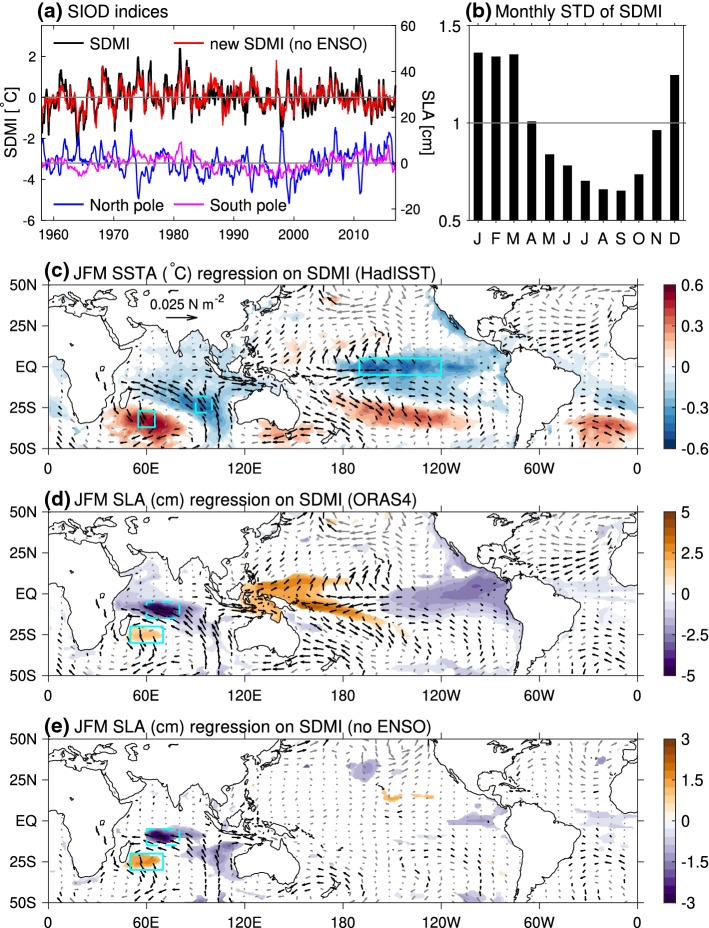
**a** Time evolution of the subtropical dipole mode index (SDMI; °C; black line), defined as the difference of SSTA from HadISST data averaged over (55°E–65°E, 37°S–27°S) and (90°E–100°E, 28°S–18°S), boxed regions in (**c**). Red line shows the SDMI independent of ENSO; blue and purple lines denote monthly SLA (cm) time series averaged in the SLA dipole areas of SCTR region in the north and Mascarene islands region in the south, respectively. Data are detrended with monthly climatology removed. **b** Monthly standard deviation of the normalized SDMI. **c** Regression of January–March (JFM) mean SSTA onto normalized JFM SDMI (°C), and vectors denote regression of the JFM surface wind stress from ECMWF twentieth-century reanalysis (N m^−2^). Box in the tropical Pacific denotes the Niño-3.4 region. **d** Same as (**c**) but for regression of JFM SLA (cm). SLA data are from ORAS4 reanalysis. Boxes in the Indian Ocean denote the SLA dipole region (60°E–80°E, 15°S–5°S and 50°E–70°E, 30°S–20°S). **e** Same as (**d**) but for regressions of SLA and wind stress residuals (with ENSO signals removed by removing linear regression on the November–January mean Niño-3.4 index) onto SDMI. Shading and black vectors in **c**–**e** denote results that are statistically significant at 90% confidence level

Over the tropical Indian Ocean, the most influential climate modes are ENSO and the Indian Ocean Dipole (IOD). The IOD is a coupled ocean–atmosphere mode operating at interannual timescale (e.g., Saji et al. 1999; Webster et al. 1999) but with significant decadal modulation (Song et al. 2007; Tozuka et al. 2007). Its positive phase is associated with cold SST anomaly (SSTA) in the tropical southeast Indian Ocean and warm SSTA in the tropical western basin, reaching peak amplitudes during September–November (Fig. 7a, b). Its temporal variability is measured by the Dipole Mode Index (DMI), which exhibits large interannual variability with decadal modulation (Fig. 7b). While some IOD events co-occur with ENSO, others are ENSO-independent (e.g., Meyers et al. 2007; Sun et al. 2015; Yang et al. 2015). Additionally, variations of monsoon winds, which are often correlated with ENSO and IOD but sometimes act independently, also induce variability in sea level over the Indian Ocean (Han et al. 2017b; Swapna et al. 2017). In the south Indian Ocean, the subtropical Indian Ocean dipole (SIOD) was identified as a coupled ocean–atmosphere mode (Behera and Yamagata 2001; Suzuki et al. 2004). Its temporal variability is measured by Subtropical Dipole Mode Index (SDMI), which peaks in January–March and is the weakest during July–September (Fig. 10b). Like the IOD, while some SIOD events are associated with ENSO, others act independently (Zhang et al. 2019).

For the multidecadal sea level trends, it is unknown to what extent the dominant effect of natural internal variability discussed above can be attributed to climate modes. For interannual variability, existing studies demonstrate the importance of ENSO and IOD in causing the observed SLAs in different coastal and island regions. Over the north Indian Ocean, the IOD-related basin-scale wind is an important cause for interannual SLAs along the coasts of Java, Sumatra and Bay of Bengal (e.g., Han and Webster 2002; Chen et al. 2016), but ENSO can also play a significant role (Sreenivas et al. 2012). Along the Indian west coast, interannual variability of boreal fall (September–November mean) sea level is correlated with the IOD (Parvathi et al. 2017), even though the magnitude is overall small there (Shankar et al. 2010; Fig. 7c, d). Over the South Indian Ocean, ENSO-related trade wind variations induce SLAs in the western tropical Pacific (Sect. 3.3), which propagate to the west coast of Australia via the ITF (e.g., Pariwono et al. 1986; Pearce and Phillips 1988; Clarke and Liu 1994; Godfrey 1996; Meyers 1996; Feng et al. 2003, 2013; Wijffels and Meyers 2004; Holgate and Woodworth 2004). Using Geosat sea level data from 1985 to 1989, Perigaud and Delecluse (1993) showed large interannual SLAs in 10°S–20°S of the Indian Ocean, with the strongest variability occurring in the El Niño year of 1986–1987.

Correlation, regression and composite analyses suggest that the IOD (ENSO) dominates interannual SLAs north (south) of ~ 10°S (e.g., Rao and Behera 2005; Yu et al. 2005; Gnanaseelan and Vaid 2010). Similar results are shown in the SLA composites for pure El Niño and pure positive IOD years from 1959 to 2008, and the ENSO-IOD co-occurrence significantly enhances the SLA magnitudes (Fig. 8; Deepa et al. 2018; also see Webster et al. 1999; Rao et al. 2002; McPhaden and Nagura 2014). Along the Indian west coast, the weak SLAs are indeed affected by IOD but also ENSO (Fig. 8). The leading EOF of SLAs over the Indian Ocean is significantly correlated with ENSO (*r* = 0.74) and IOD (0.62) during 1993–2016 (Fig. 7c, d). The somewhat higher correlation with ENSO is likely due to the inclusion of the entire tropical South Indian Ocean where ENSO’s influence is strong. The effect of monsoon wind variability that is independent of ENSO and IOD on interannual coastal SLAs requires systematic investigation.

On decadal time scales, the impacts of climate modes have strong seasonality and spatial variability (Han et al. 2018). During boreal winter, climate modes account for most observed decadal SLAs from 1962 to 2011, with the total effects of IPO, IOD and monsoon accounting for 86%, 84%, 80% and 78% of the observed SLA standard deviation (STD) near the Australian coast, SCTR region, coasts of Sumatra and coasts of the eastern-northern Bay of Bengal, respectively. During summer, they explain 95% of observed STD near the Australian coast but only 67%, 58% and 63%, respectively, over the SCTR, Sumatra and Bay of Bengal coasts. In the equatorial and North Indian Ocean, wind associated with IPO is the major cause for coastal SLAs along the coasts of Java, Sumatra and Bay of Bengal during winter. However, during summer, winds associated with decadal IOD and to a lesser degree monsoon control the coastal SLAs, through both remote forcing from the equator and local forcing adjacent to the coasts (Han et al. 2018). In the South Indian Ocean, IPO dominates SLAs near the Australian coast and, to a lesser extent, the SCTR region (Frankcombe et al. 2015; Deepa et al. 2019) during both winter and summer (Han et al. 2018), with a sea level fall in the SCTR islands region corresponding to a SLR along the boundaries of the East Indian Ocean (Fig. 9A, B). The trade wind anomalies in the tropical Pacific associated with the IPO are the major cause for decadal SLAs along the Australian west coast via the ITF (Feng et al. 2004, 2010; Behera and Yamagata 2010), with local winds, which are partly associated with the IPO (Han et al. 2018) playing a comparable role (Trenary and Han 2013). Over the SCTR region, IPO drives the SLAs through affecting the surface wind over the Indian Ocean, with the influence of ITF being negligible (Deepa et al. 2019; Han et al. 2018). Decadal variations of winter and summer monsoon winds also have significant contributions, and the effect of IOD is non-negligible during winter (Fig. 9A).

The impact of SIOD on interannual SLAs shows a dipole structure over the southwest basin and its influence is negligible along the eastern and northern Indian Ocean boundaries (Fig. 10). A positive SIOD corresponds to negative SLA over the Chagos Archipelago of the SCTR and positive SLA over the Mascarene islands (e.g., Réunion, Mauritius and Rodrigues) southeast of Madagascar. Therefore, the SIOD primarily affects sea level of the island nations of the southwest Indian Ocean. The basin-scale wind stress curls associated with the SIOD excite Rossby waves propagating from the east; combined with forcing by the local wind stress curl, they produce the interannual SLA dipole, which also exhibits significant decadal variations (Zhang et al. 2019).

### Western Indian Ocean Boundary

#### Remote Versus Local Processes

Compared to the eastern and northern boundaries, studies on sea level variability along the western boundary (African coastline) are lacking, likely due to the relatively weaker SLA amplitude and shorter tide gauge records there. Along the east coasts of South and North Africa, multidecadal trend and interannual-to-decadal SLAs seem to be connected to the open-ocean SLAs with substantially reduced magnitudes (Figs. 6, 7c and 9), indicating that part of the open-ocean Rossby waves’ energy may pass the slope barrier (Sect. 2) to arrive at the western boundary. Near Zanzibar (Fig. 6), the continental slope is steep and the shelf is narrow; therefore, signals from the open ocean could have a significant impact on the observed SLA, including the observed falling trend there (see Sect. 2).

#### Effects of Climate Modes

While both ENSO and IOD are associated with interannual and decadal SLAs in some areas of the western boundary including the coast of Madagascar (Figs. 7, 8, 9), and the SIOD is associated with weak SLAs along the Somali coast (Fig. 10), variations of monsoon appear to have an overall larger effect on decadal SLAs for both winter and summer (Figs. 9A, B). How the Rossby waves generated in the basin interior affect the Somali current, East African coastal current and western boundary SLAs remains unknown. How the climate modes affect sea level in the Persian Gulf and Red Sea remains unclear.

## The Atlantic Ocean

Different from the Pacific and Indian Oceans whose coastal sea level variability is dominated by the wind-driven ocean circulation, sea level along the Atlantic coasts—particularly the east coast of North America—may also be influenced by the Atlantic Meridional Overturning Circulation (AMOC) driven by buoyancy flux, as suggested by OGCM and coupled climate model studies. Observational evidence, however, has not yet firmly established the relationship between AMOC and coastal sea level variability.

### Observations

**Fig. 11:**
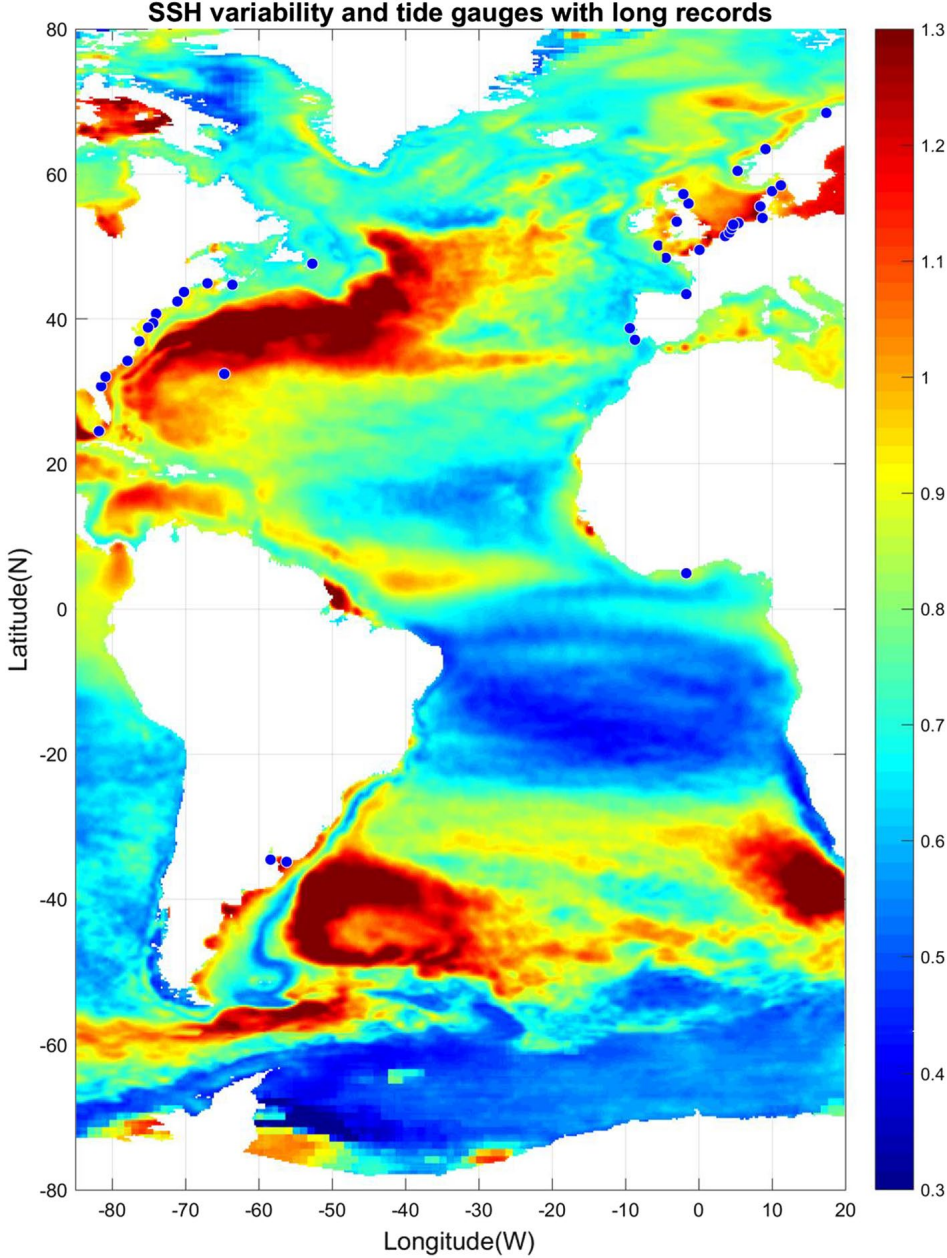
Sea surface height anomaly (SSHA) (units are log of root mean square in cm) from satellite altimeter data (1993–2017) and location of tide gauge stations (blue dots) with records of 75 years and longer and no major gaps. Altimeter data are from the Permanent Service for Mean Sea Level (PSMSL) organization (https://las.aviso.oceanobs.com/) and tide gauge data are from the Permanent Service for Mean Sea Level (PSMSL) organization (http://www.psmsl.org/)

**Fig. 12:**
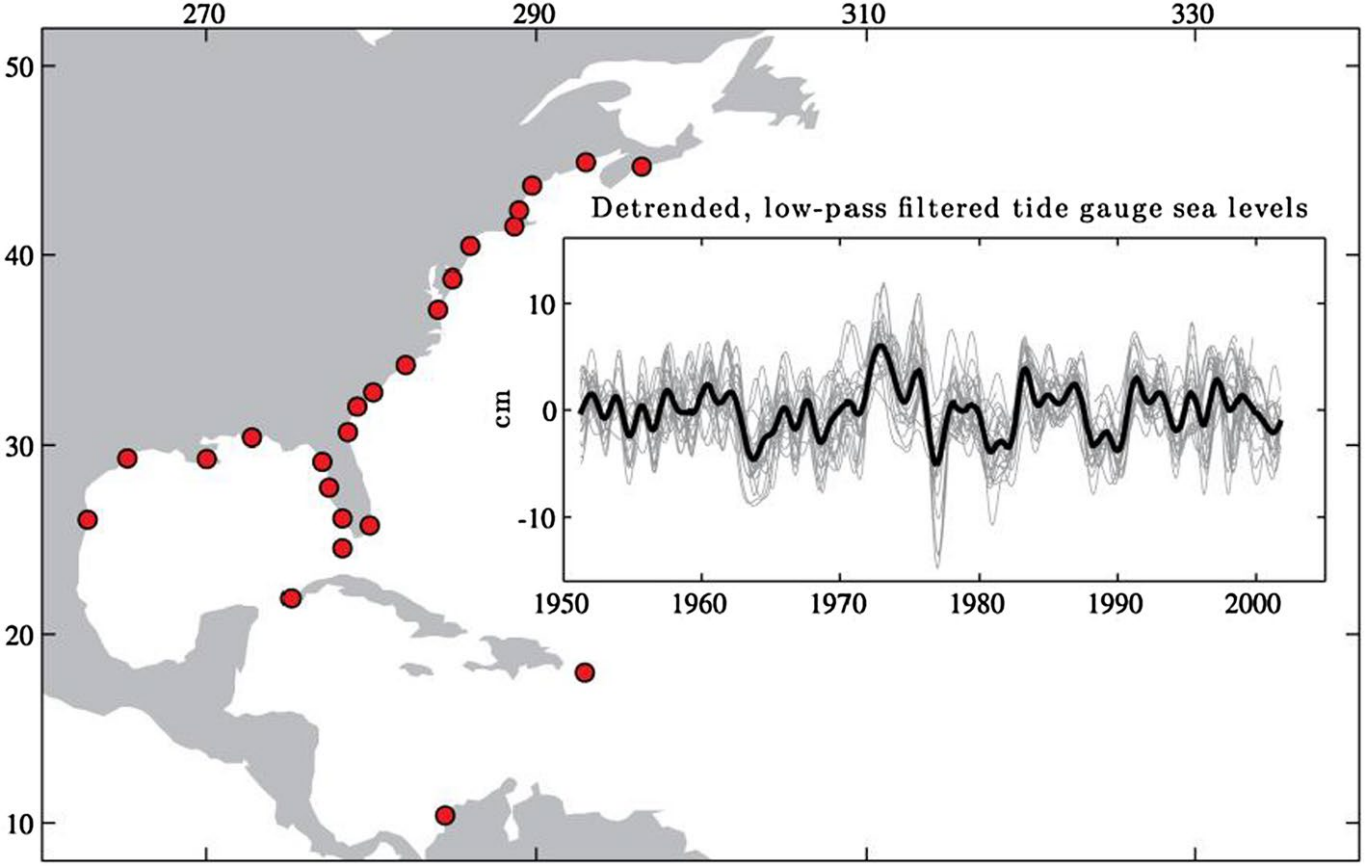
Tide gauge locations (red circles) and the detrended, 18-month low-pass filtered sea level anomaly time series (gray lines) with monthly climatology removed at each tide gauge, based on the PSMSL monthly tide gauge observations from 1950 to 2010. The thick black line is the average of the individual time series and accounts for greater than 50% of the variance in the low-passed data set. The filter passed more than 90% of the amplitudes at periods longer than 1.9 years, and thus the SLA represents interannual-to-decadal variability. From Thompson and Mitchum (2014)

The spatial distribution of sea level variability in the Atlantic as seen in altimeter data from 1993 to 2017 (Fig. 11) identifies several regions of large-amplitude SLAs, most notably the Gulf Stream in the western North Atlantic, the Brazil Current in the western South Atlantic, the South African coast in the eastern South Atlantic where the Agulhas rings shed from the Indian Ocean, and the North Sea as well as other European shelf regions of the eastern North Atlantic. Tide gauge records also show large-amplitude interannual and decadal SLAs along the European coast, and to a lesser extent the east coast of North America (Figs. 1 and 12). Spectral peaks at interannual, 12–14 year, bidecadal 20–30 year and 50–70 year periods were detected in most of the tide gauges along the US northeast coast (e.g., Figure 1 of Kenigson and Han 2014; Sects. 5.2 and 5.3), and the near 60-year cycle was also observed along European coasts and other ocean basins (Chambers et al. 2012).

Using long-tide gauge records, several recent studies detected a region of rapid SLR acceleration along the US northeast coast from Cape Hatteras to Cape Cod during recent decades particularly in the ~ 1990–2010 period (e.g., Boon 2012; Ezer and Corlett 2012; Sallenger et al. 2012). Sallenger et al. (2012) dubbed the region of SLR rate much faster than the global mean, from Cape Hatteras to Cape Cod, the “hotspot.” From 2010 to 2015, however, the area of the highest SLR rate shifts to south of Cape Hatteras, suggesting interannual and decadal variability of the rapidest SLR region (Valle-Levinson et al. 2017; Domingues et al. 2018; Ezer 2019).

Long-tide gauge records with lengths ≥ 75 years and no major gaps (blue dots of Fig. 11) are available primarily along the west and east coasts of the North Atlantic, with only a few available along the coasts of the South Atlantic. Nevertheless, coherent interannual SLAs were also detected by tide gauges along the South Atlantic west and east coasts, respectively (Brundrit et al. 1984, 1987; Douglas 2001; Papadopoulos and Tsimplis 2006). Based on the data availability and dynamical distinctions, in our discussion below we divide the Atlantic into three regions: the western North Atlantic, the eastern North Atlantic and the South Atlantic. Each region is not only unique in the dynamics involved, but also in the amount of data available (Fig. 11), leading to substantial differences in our understanding of the relevant processes and time scales of variability.

### Western Boundary of the North Atlantic

#### Remote Versus Local Processes and Buoyancy Versus Wind-Driven

Sea level along the US east coast has received renewed attention in recent years, due to the detection of the accelerated SLR in the mid-Atlantic Bight (Sallenger et al. 2012; Ezer 2013; Ezer et al. 2013; Yin and Goddard 2013; Goddard et al. 2015; Ezer 2015). While the IB effect was suggested to contribute 10–30% of the acceleration (Piecuch and Ponte 2015), a slowdown of the AMOC was suggested to be an important cause (Sallenger et al. 2012), based on a qualitative comparison between the observed coastal SLR pattern with that of climate model experiments driven by buoyancy flux of Yin et al. (2009). Extensive modeling studies showed that surface warming and freshwater flux into the subpolar North Atlantic increased steric sea level, weakened the AMOC, reduced the sharp sea level gradients across the Gulf Stream and North Atlantic Current, and resulted in rapid dynamical SLR along the northeast coast of North America (e.g., Hakkinen 2001; Hakkinen and Rhines 2004; Levermann et al. 2005; Hewitt et al. 2006; Landerer et al. 2007; Kleinen et al. 2009; Yin et al. 2009; Lorbacher et al. 2010; Hu et al. 2011; den Toom et al. 2014; Brunnabend et al. 2015; Yu et al. 2016; Hu and Bates 2018). Because the mean sea level is low on the inshore side but 1–1.5 m higher on the open-ocean side of the Gulf Stream, a weakened cross-stream gradient corresponds to a rising sea level inshore and falling sea level offshore of the Gulf Stream; these signals are clear in climate model solutions (e.g., Yin et al. 2009).

On interannual timescales, OGCM experiments showed an anticorrelation between the US northeast coast SLAs and variability of the Atlantic meridional overturning circulation (AMOC) strength, with a 2 cm drop in sea level corresponding to a 1 Sverdrup (Sv) increase in the AMOC (Bingham and Hughes 2009). It is unclear, however, whether this anticorrelation represents the effect of buoyancy-driven geostrophic component of the AMOC or reflects large-scale wind-driven ocean circulation. Note that the northward transport of AMOC’s upper branch consists of three components: the Gulf Stream (or at higher latitudes, the North Atlantic Current), meridional Ekman transport driven by wind and upper-mid-ocean transport driven by wind and buoyancy flux in the open ocean (McCarthy et al. 2015; Ezer 2015; Piecuch et al. 2019).

Using the RAPID monitoring array at 26°N and tide gauge data along the New England coasts during 2004–2017, Piecuch et al. (2019) found that the anticorrelation between intraseasonal/interannual coastal SLAs and AMOC transport was forced by large-scale wind patterns. While the local longshore winds drive negative coastal SLAs, open-ocean winds along 26°N force positive (northward) Ekman transport, resulting in an anticorrelation between SLAs and the AMOC. The geostrophic component of AMOC (Gulf Stream plus upper-mid-ocean transport), however, is uncorrelated with coastal SLAs. In fact, correlations of 0.70–0.95 are shown between the Ekman component of AMOC transport and three interannual modes from empirical mode decomposition (EMD) of the SLA difference between Atlantic City and Bermuda, using the RAPID data and tide gauge observations during 2004–2012; the correlations are low between the geostrophic AMOC and SLA difference except for the ~ 6-year EMD mode, which shows a correlation of 0.89 but it only has one cycle during the 2004–2012 period (Fig. 8 of Ezer 2015; also see Ezer et al. 2013). Wind stress curl in the Labrador Sea shows significant correlation with interannual SLAs along the US northeast coast (Andres et al. 2013; Kenigson et al. 2018), and IB effect accounts for ~ 25% in the region (Piecuch and Ponte 2015). These results agree with Piecuch et al. (2019), suggesting that the anticorrelation between AMOC and US northeast coast SLAs on interannual timescales during the RAPID period are primarily wind-driven. Further studies are needed to explore the relationship between geostrophic AMOC and US east coast interannual SLAs using observations.

Modeling solutions support the above observational results. The local longshore winds (Woodworth et al. 2014) are shown to be important for interannual SLAs all the way from Cape Hatteras to Nova Scotia (Andres et al. 2013; Kenigson et al. 2018). Using a global barotropic model, Piecuch et al. (2016) showed that local wind stress over the continental shelf and slope is the major forcing for interannual SLAs along the coast north of Cape Hatteras from 1980 to 2015, explaining 50% of tide gauge variance.

On decadal timescales, modeling studies showed that variations of both surface buoyancy flux and wind stress over the North Atlantic can induce AMOC variability (e.g., Marshall et al. 2001; Schloesser et al. 2012, 2014; Yeager and Danabasoglu 2014; Danabasoglu et al. 2016; Elipot et al. 2017). OGCM experiments, however, suggested that changes of downward heat flux were the major cause for the spin down/up in the Subpolar Gyre and AMOC, causing the 12–14 year SLAs along the US northeast coast (Hakkinen 2000, 2001; Hakkinen and Rhines 2004), with 1 Sv weakening of AMOC corresponds to 1.5 cm SLR along the US northeast coast (Woodworth et al. 2014). Local longshore winds may also affect the decadal SLAs (Woodworth et al. 2014). Due to the short data record of the RAPID array, observational relationship between geostrophic AMOC and US east coast SLAs on decadal timescales has not yet been established.

South of Cape Hatteras, interior wind stress curl is the dominant forcing for coastal SLAs at interannual-to-decadal time scales (e.g., Sturges and Hong 1995; Ezer 1999; Hong et al. 2000; Woodworth et al. 2014). Baroclinic Rossby waves generated by open-ocean wind stress curl propagate westward, affecting the Gulf Stream transport, cross-stream sea level gradient and thus coastal sea level (Hong et al. 2000; Sect. 2). Both wind stress curl and surface heat flux in the basin interior could cause interannual-to-decadal variability of the Gulf Stream (e.g., Curry and McCartney 2001; Di Nezio et al. 2009; Meinen et al. 2010; Chaudhuri et al. 2011), but these studies did not explicitly examine coastal sea level. Indeed, data analyses and modeling experiments showed significant anticorrelations between the Gulf Stream transport and US east coast SLAs on a wide range of time scales, from a few days to decades (Hong et al. 2000; Ezer 2001; Sturges and Hong 2001; Papadopoulos and Tsimplis 2006; Ezer 2013; Ezer et al. 2013; Kopp 2013; Ezer and Atkinson 2014, 2017; Park and Sweet 2015).

It has also been argued that variations in the Gulf Stream position are related to variations in the southward-flowing Slope Current in the mid-Atlantic-Bight (Rossby et al. 2010), which connects the recirculation gyre between the coast and the Gulf Stream. When the Gulf Stream moves offshore, the northern recirculation gyre between the Gulf Stream and the coast can expand and the Slope Current along the coast can increase its flow, resulting in increased coastal sea level (Ezer et al. 2013; Ezer 2015, 2019). Conversely, other studies suggest that intrusion of the Labrador Sea Water into the Slope Sea affects coastal sea level gradients and therefore the latitudinal position and separation point of the Gulf Stream (e.g., Rossby 1999; Hameed and Piontkovski 2004; Andres et al. 2013).

Despite the dynamical differences between coastal regions north and south of Cape Hatteras, there are multidecadal periods when coherent SLAs were observed from the Caribbean to Nova Scotia (e.g., Maul and Hanson 1991; Hakkinen 2000; Douglas 2005; Thompson and Mitchum 2014; Calafat et al. 2018; Fig. 12). This coherence is surprising given the substantial longshore distances and the distinct dynamical regimes partitioned by the Gulf Stream separation and the Straits of Florida. Analyses of ocean hindcasts find that the spatial coherence is largely the result of changes in upper ocean heat content spanning the coastal and open-ocean areas of the western boundary region rather than changes in slope across the boundary current (Hakkinen 2000, Thompson and Mitchum 2014). During a large part of the second half of the twentieth century, the spatial coherent coastal SLAs are largely driven by changes in basin-wide wind stress curl that force basin-scale, zonal redistributions of volume into and out of the western boundary region (Thompson and Mitchum 2014). It has also been shown that Rossby waves have coherent modulation on the amplitudes of sea level annual cycle along US east coast (Calafat et al. 2018).

#### Effects of Climate Modes on US East Coast Sea Level

Two climate modes dominate the variability of the North Atlantic Ocean: the North Atlantic Oscillation (NAO)—representing variations in the amplitude of a dipole pattern in sea level pressure over the Atlantic (e.g., Barnston and Livezey 1987; Hurrell 1995)—and the Atlantic Multidecadal Oscillation (AMO)—representing the average SST over the North Atlantic (e.g., Schlesinger and Ramankutty 1994; Delworth and Mann 2000; Kerr 2000; Enfield et al. 2001a, b). Both have imprints in surface wind stress and buoyancy flux and thus are closely connected with variations in gyre strength and AMOC (e.g., Curry and McCartney 2001; Schloesser et al. 2014). The NAO and AMO have different time scales, with more interannual-to-decadal variability in NAO compared to ~ 60–80-year time scales of the AMO (Delworth and Mann 2000). It has been hypothesized that at multidecadal timescales the SST variability of the AMO reflects the temporal integration of the ocean circulation (and associated heat transport) variability forced by wind stress associated with the NAO (McCarthy et al. 2015; Woodworth et al. 2016); therefore, NAO and AMO may not be fully independent of each other. It has also been argued that variations of the AMO SST index in the past few decades resulted partly from external forcing (natural and anthropogenic; e.g., Booth et al. 2012; Knudsen et al. 2014; Mann et al. 2014) and partly from natural variability (Ting et al. 2009; Zhang et al. 2013). In addition, a recent study suggested the influence of ENSO on multidecadal variability of US east coast sea level (Valle-Levinson et al. 2017).

**Fig. 13:**
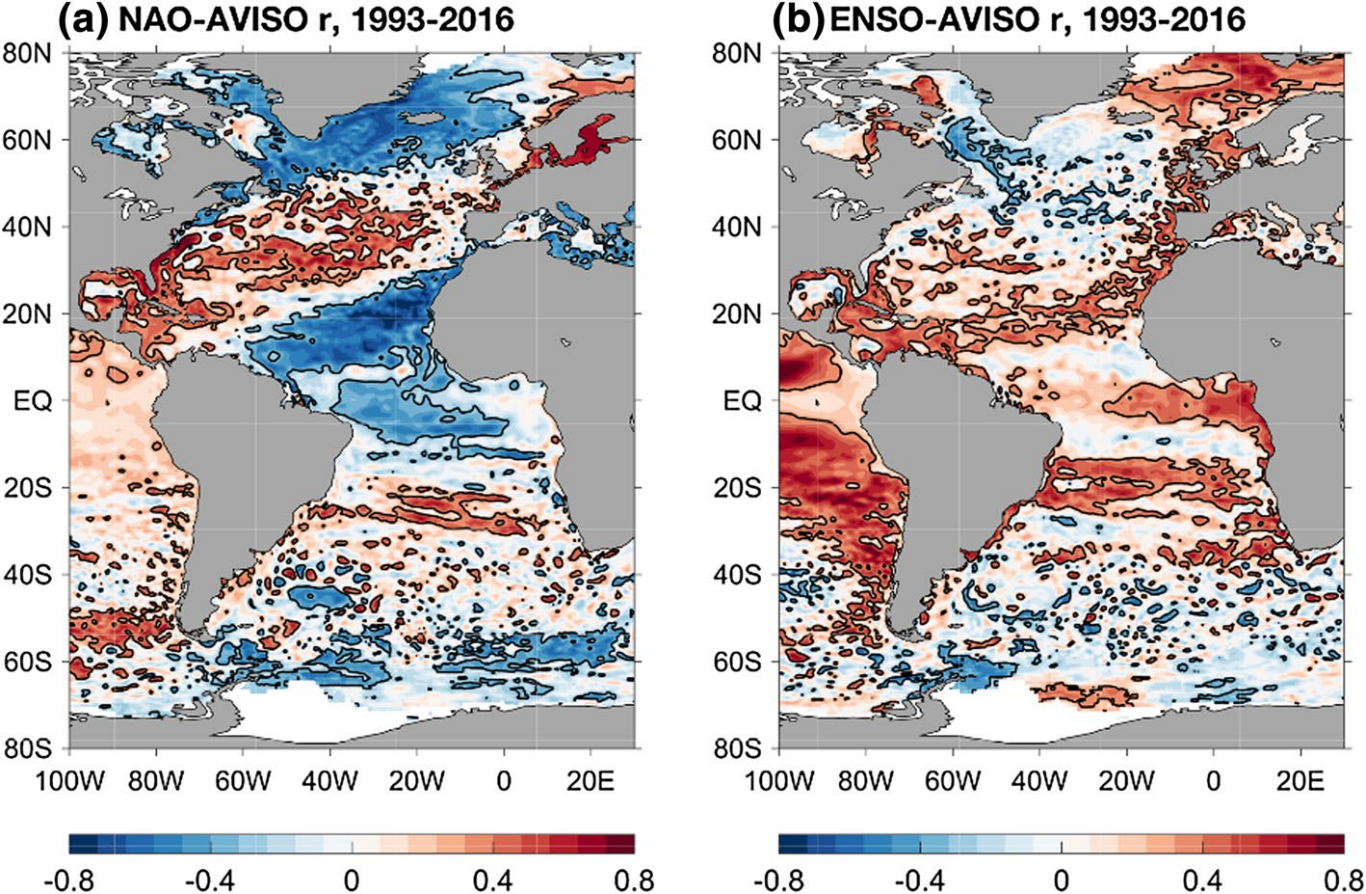
**a** Correlation map between winter (DJFM mean) NAO index and annual mean AVISO satellite SSHA over the Atlantic Ocean for the 1993–2016 period; correlation coefficients of ± 0.3 are shown by the black line contours; **b** Same as **a** but for the correlation between ENSO and satellite SSHA. (The NAO index is downloaded from: www.climatedataguide.ucar.edu/climate-data/hurrell-north-atlantic-oscillation-nao-index-station-based.)

**Fig. 14:**
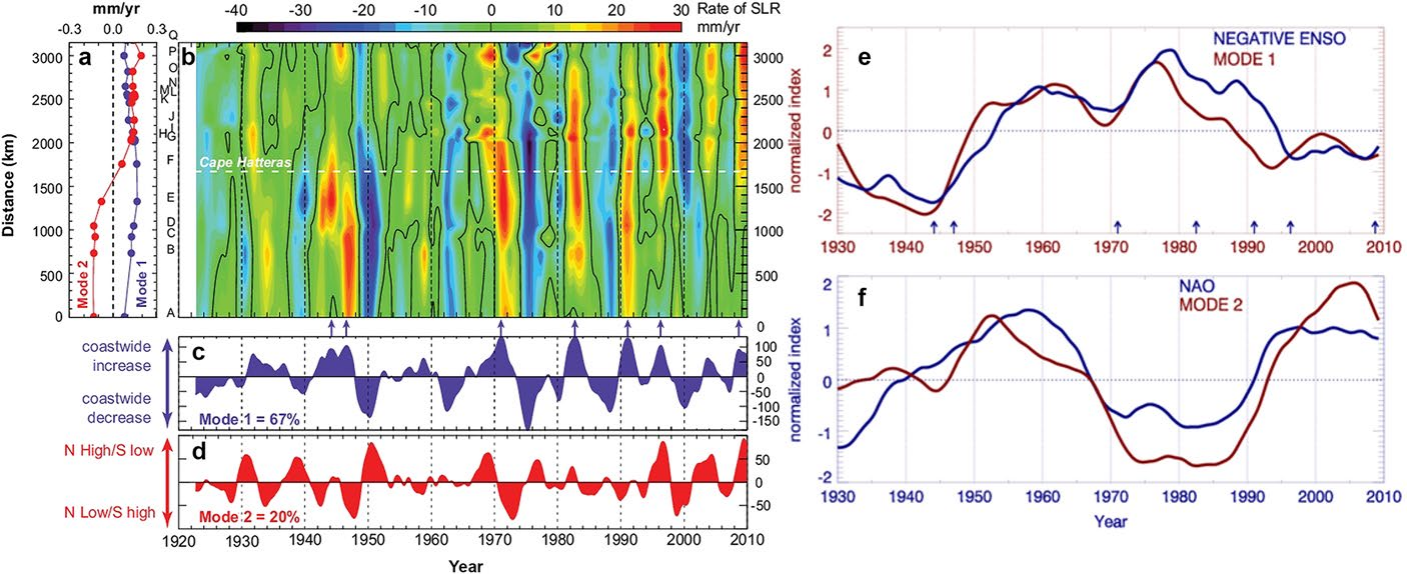
The 5-year rates of sea level change (in mm/yr) calculated from the detrended, 1-year filtered data for the long time series of tide gauge along the US east coast from 1920 to 2010. **a** Spatial structures of the first two EOF modes; **b** Hovmöller diagram of the sea level change rate along US east coast from tide gauge A (southern tip of Florida) to Q (Maine); the white-dashed horizontal line shows the location of Cape Hatteras; **c**, **d** time series of the principle components (or temporal variability) of EOF modes 1 and 2 shown in **a**. The blue arrows above **c** show that the highest peaks in Mode 1 correspond to the timing of the SLR hot spots in **b**. Cumulative and 7-year filtered versions of **e **EOF Mode 1 and negative ENSO index and **f ** EOF2 and NAO index. The blue arrows are like those between panels (**b**) and (**c**). Customized from Valle-Levinson et al. (2017)

The surface wind and heat flux, which drive interannual and decadal SLAs along the west coast of North Atlantic through both remote and local processes (Sect. 5.2.1) are in part associated with the NAO. Satellite observations from 1993 to 2016 show that the NAO is associated with distinct spatial patterns of coastal SLA (Fig. 13), showing opposite SLA signs between north and south of Cape Hatteras with lower (higher) sea level in the north (south) during a positive NAO. Tide gauge records from 1920 to 2010 confirm this north–south contrast pattern, which appears in the 2nd EOF mode of coastal SLAs (Fig. 14a, red line). This EOF2 mode however is not significantly correlated with the NAO index using 1 year filtered data (Valle-Levinson et al. 2017), which is inconsistent with the correlation shown in Fig. 13; but the 7 year filtered cumulative EOF2 and NAO index agree well (Fig. 14f), showing coherent 50–70 year cycle. This result indicates that cumulative ocean response to NAO forcing can produce ~ 60 year cycle in coastal SLA.

The inconsistency of correlations between coastal SLA and NAO using satellite data of 1993–2016 and tide gauge data of 1920–2010 indicates that NAO impact may exhibit significant decadal variability. Empirical analyses showed that the NAO impact on interannual SLAs is strong during some decades but weak during others (e.g., Woolf 2003; Andres et al. 2013; Woodworth et al. 2017), due to changes of local longshore wind associated with changes in NAO-related wind patterns near the coast (Kenigson et al. 2018). A strong negative NAO superimposed on a 30% AMOC reduction likely caused the 2009–2010 extreme SLR along the US Northeast coast (Goddard et al. 2015; Ezer 2015), with up to 50% of the sea level increase resulted from the IB effect (Piecuch and Ponte 2015).

Tide gauge data showed that the ~ 60-year cycle in US northeast coast sea level coincided with the positive transition of the AMO index, with SST warming since ~ 1970 corresponding to rapid SLR in the mid-Atlantic Bight (Kopp 2013; Ezer 2013; Ezer et al. 2013; Scafetta 2014). Kenigson and Han (2014) demonstrated that the ~ 60-year cycle has contributed a large portion to the SLR acceleration along the US northeast coast since ~ 1970; without the ~ 60-year cycle, the acceleration dramatically reduces. Empirical analyses indicated that the US northeast coast SLR acceleration is also linked to the sea level pressure and winds associated with the NAO (e.g., Ezer 2015). Tide gauge data during the past 95 years showed that the locations of accelerated SLR exhibit significant intra-decadal variability, and they were not correlated with the AMO (Valle-Levinson et al. 2017). While the multidecadal variation of the spatially coherent SLAs along the entire US east coast (EOF1 of Fig. 14a, black line) agrees well with the 7-year filtered cumulative ENSO index (Fig. 14e), the EOF2 mode, which shows opposite signs north and south of Cape Hatteras, corresponds to cumulative NAO index. They suggested that the cumulative ocean response to the combined ENSO and NAO forcing determines the timing and location of the accelerated SLR, even though direct correlations for EOF1-ENSO (also see Fig. 13b) and EOF2-NAO were not found. Near the coasts of Caribbean and Gulf of Mexico, ENSO has higher correlations with coastal SLAs (Fig. 13b).

### The Eastern Boundary of the North Atlantic Ocean

#### Remote Versus Local Processes

Along the east coast of the North Atlantic, while some studies suggested the likely link between coastal SLAs and changes of gyre-scale ocean circulation (Miller and Douglas 2007; Woodworth et al. 2010), others show the dominance of remote and local coastal processes, rather than remote forcing from the basin interior, are the primary causes for coastal SLAs (e.g., Sturges and Douglas 2011; Calafat et al. 2012; Wahl et al. 2013; Dangendorf et al. 2014; Chafik et al. 2019). Interannual and decadal SLAs at Cascais tide gauge, Portugal (39°N, 9°W), was primarily forced by longshore wind integrated from the equator to Cascais, suggesting the dominant role played by northward propagation of coastally trapped waves driven by longshore wind (Sturges and Douglas 2011). Along the European west coast and in the Mediterranean Sea (27°N–55°N), decadal SLAs up to 15 cm in tide-gauge data are highly correlated with the cumulative longshore wind stress, which further demonstrates the deterministic role of coastally trapped waves forced by longshore wind (e.g., Calafat et al. 2012; Chafik et al. 2019); the IB effect contributes 14–27% sea level variance and forcing by local wind and heat flux is weak (Calafat et al. 2012). These coastal SLA signals can propagate into the Norwegian coast, Barents Sea and Kara Sea, affecting coastal sea level there (Richter et al. 2012; Calafat et al. 2013). In the Mediterranean Sea, coastal sea level variability results from the IB effect, surface wind forcing and mass exchange with the Atlantic Ocean, and mass exchange dominates other factors during some years such as 2010 (e.g., Fukumori et al. 2007; Menemenlis et al. 2007; Gomis et al. 2008; Calafat et al. 2012; Landerer and Volkov 2013; Tsimplis et al. 2013; Volkov 2019). South of 27°N, decadal SLAs along the African west coast of the North Atlantic are associated with heat advection by Ekman transport (Calafat et al. 2012).

In the North Sea, various processes account for interannual SLAs detected by tide gauge data around the coasts (Dangendorf et al. 2014). While the IB effect dominates the SLAs along the UK and Norwegian coasts, wind controls the SLAs in the south from Belgium up to Denmark. On decadal time scales, SLAs mainly reflect steric changes and result largely from remote longshore wind forcing. Spatial correlation analyses of altimetry observations and steric heights suggest coherent SLAs extending from the Norwegian shelf in the north down to the Canary islands of Spain in the South, further supporting the importance of longshore wind-forced coastally trapped waves.

#### Effects of Climate Modes

Since the NAO index is based on sea level pressure pattern west of the European coast, a strong influence from NAO on sea level via changes in wind stress and sea level pressure (IB effect) is expected. Numerous studies find that IB effect and large-scale wind patterns that drive the coastal SLAs along the eastern boundary of the North Atlantic spanning the coasts of Morocco, Europe and Mediterranean Sea, largely result from the NAO (e.g., Wakelin et al. 2003; Woolf et al. 2003; Yan et al. 2004; Hughes and Meredith 2006; Tsimplis et al. 2006; Miller and Douglas 2007; Gomis et al. 2008; Tsimplis and Shaw 2008; Calafat et al. 2012; Tsimplis et al. 2013; Dangendorf et al. 2014; Ezer et al. 2016). The NAO impacts, however, are spatially and temporally variable, with stronger NAO influence observed during the second half of the twentieth century relative to the first half. Spatial variations of the NAO influences are evident, with positive coastal SLA-NAO correlations appearing in the North Sea and northern coasts of Europe, but negative correlations in the south as revealed by satellite altimetry data from 1993 to 2016 (Fig. 13a). Changes in the center of action of the Icelandic low and Azores high associated with the NAO, which affect surface wind, sea level pressure and SST patterns, play a crucial role in affecting decadal sea level variability and multidecadal trend along both the east and west coasts of the North Atlantic Ocean (Kolker and Hameed 2007).

**Fig. 15:**
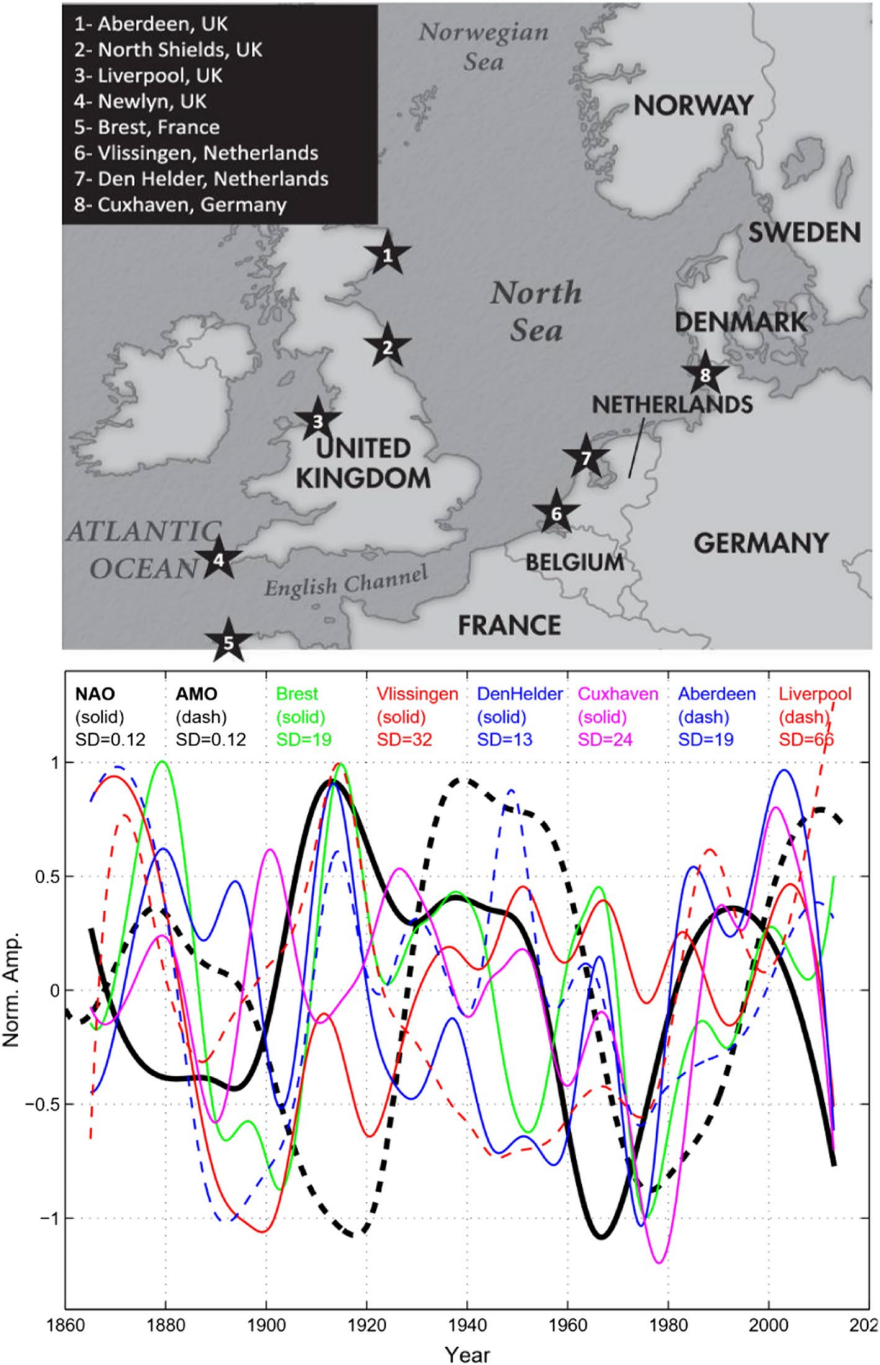
(Top) Location of tide gauge stations near European coast; (Bottom) low-frequency modes of SLA (colored, thin lines) obtained from Ensemble Empirical Mode Decomposition (EEMD) method and indices of NAO (solid, heavy, black line) and AMO (dashed, heavy, black line) for 1865–2012 at six of the eight tide gauge stations shown in the top panel; the amplitude is normalized by the standard deviation (SD), which is listed for each record (NAO and AMO have no units; sea level is in mm). Adapted from Ezer et al. (2016)

The decadal variations of coastal sea level along the coasts of the North Sea and the UK can be either dominated by low-frequency variations of the NAO or AMO, depending on the temporal periods (Ezer et al. 2016). The ~ 60-year cycle of AMO is evident in Fig. 15 with negative AMO phases in ~ 1910–1920 and ~ 1970–1980 and positive AMO phases in ~ 1930–1950 and ~ 2000–2012; however, the very low-frequency variations of the NAO are in different phases. Around 1915–1920 when NAO is positive and AMO is negative, five of the six stations show positive SLA maxima indicating that NAO may dominate the sea level variability (i.e., the positive correlation in Fig. 13a), while around 1980 when AMO is negative and NAO is near zero, all 6 stations show peak negative SLAs indicating that AMO is likely dominating the SLAs. The bidecadal variations of tide-gauge observed SLAs along the east and west coasts of the North Atlantic were also shown to be associated with the AMO (Frankcombe and Dijkstra 2009). In addition, ENSO also seems to be correlated with SLAs near the eastern boundary of the North Atlantic (Fig. 13b).

### The Western and Eastern Boundaries of the South Atlantic Ocean

#### Remote Versus Local Processes

Due to the very limited long-term tide gauge records in the South Atlantic Ocean, studies of remote influence on coastal sea level are rare and often involve numerical model simulations rather than observations. The few analyses of tide gauge data, such as studies of sea level trends at the southernmost tip of South America (e.g., Dragani et al. 2014), show significant interannual variations, but the lack of long-term records prevents researchers from fully understanding the sources of this variability. In the western South Atlantic, circulations near the coast of South America are largely influenced by local winds and tides (e.g., Tonini and Palma 2011). Modeling studies suggest that the upwelling dynamics of the shallow coastal area near the Brazilian coast are largely influenced by a combination of local winds and the interaction of the Brazil Current with the coastal topography (Palma and Matano 2009). While this study focused on seasonal upwelling, one may expect that longer-term variations in the wind and the Brazil Current would also impact the coastal sea level. Observations by Olson et al. (1988) found that the Brazil Current as well as the Malvinas (Falkland) Current (a cold current flowing northward along the tip of South America opposing the Brazil Current) have significant interannual variations in their separation point from the coast, which can thus cause interannual variations in coastal sea level. No studies are available that examine the forcing mechanisms of coastal sea level variability.

Piecuch and Ponte (2013) used models to study interannual SLAs in the tropical South Atlantic Ocean. They showed that Rossby waves forced by surface wind and buoyancy fluxes communicated across the basin in a similar fashion to processes found in other tropical and subtropical regions. By analyzing ocean reanalysis data, Vianna and Menezes (2013) showed that the leading Complex EOF mode of the bidecadal sea level signals over the entire Atlantic basin is characterized by in phase variations of Subtropical Gyres in the North and South Atlantic with an opposite sign in the tropical and subpolar regions, and it is associated with the AMOC variability. For the second CEOF mode, the North and South Subtropical Gyres are not in phase, and it is associated with westward propagation of temperature anomalies via Rossby waves (also see Sevellec and Fedorov 2013). The impacts of AMOC and Rossby waves on coastal sea level along the South American coasts, however, remain unclear. Their interactions with the western boundary current, the southward-flowing Brazil Current (maximum variability around ~ 50°W, 40°S; Fig. 11) and thus coastal SLAs are not known. One of the few observations of AMOC in the South Atlantic is the 35°S section from Africa to South America (Dong et al. 2009), which shows seasonal and interannual variations that impact variations in the northward heat transport. These observations also indicate large interannual variability in the eastern and western boundary currents, but the influence of these variations on coastal sea level has not been evaluated.

Along the eastern boundary of South Atlantic (i.e., west coast of Africa), remote influence from the equatorial Atlantic by events similar to the Pacific El Niño can cause coherent interannual sea level variability of up to 10–20 cm along the entire coast (Brundrit et al. 1984, 1987). Years of 1963, 1974 and 1984 are the years similar to Pacific El Niño, causing SLR along the west African coast. Although the modeling work of Piecuch and Ponte (2013) focused on the open ocean, their results suggest the importance of wind forcing versus effects of buoyancy, ocean internal variability and nonlinear ocean response in causing interannual SLAs near the west coast of Africa (their Fig. 2). The effects longshore winds versus equatorial forcing, local versus remote longshore wind and buoyancy versus wind, however, have not been examined. The influence of northward flowing Benguela Current along the western coast of Africa is not known. In the southeast Atlantic basin, energetic rings shed from the Agulhas Current over the southern tip of Africa (e.g., Olson and Evans 1986) propagate northwestward and contribute to considerable sea level variability there (~ 10°E, 40°S; Fig. 11). These rings are generally larger than Gulf Stream eddies, and they are shed in an irregular frequency with a signature clearly seen in satellite altimetry data (e.g., Schouten et al. 2000; review of Beal et al. 2011). The impact of the rings on coastal sea level has not been studied, however.

#### Effects of Climate Modes

Existing studies found that the AMOC in the South Atlantic is correlated with SST, which leads to a reconstruction of South Atlantic AMOC (Lopez et al. 2017); this study shows a strong connection between the South Atlantic AMOC, SST and the IPO. Therefore, decadal variations in the South Atlantic Ocean are expected to be quite different from those in the North Atlantic Ocean, which are largely influenced by the NAO. ENSO has significant impacts on coastal SLAs along the southwest Atlantic coast (*r* = 0.5 for SLA and SOI index from 1958 to 1997) and the correlation increased after 1980, likely through the Pacific–South American teleconnection mechanism by affecting surface wind, sea level pressure and precipitation (Papadopoulos and Tsimplis 2006; also see Douglas 2001). While ENSO is significantly correlated with SLAs at tide gauge Buenos Aires, east coast of Argentina within the estuary, from the late 1910 to early 1980s, the SLAs at tide gauge Quequen, which is somewhat south of Buenos Aires near the open ocean, are not correlated with ENSO (Douglas 2001). This result further suggests the complication of coastal processes relative to open-ocean sea level.

Correlation analyses using satellite altimetry data from 1993 to 2016 show weak influence of NAO on south Atlantic coastal sea level except for some areas along the southeast coast of South America (Fig. 13a), but ENSO appears to have significant effects near the coasts of Caribbean Sea, and the north as well as some regions of the southeast coast of South America (Fig. 13b). Comprehensive studies using both observations analysis and modeling experiments are needed to understand the climate modes impact on coastal sea level at various time scales in the South Atlantic Ocean.

## Summary, Issues and Future Outlook

### Summary

While reviewing coastal sea level variability and underlying mechanisms, one has to emphasize on the significant impacts of climate modes on coastal oceans via both local and remote processes, such as sea level signals from the open ocean. Tide gauge data detected large interannual and decadal sea level variability around the coasts of the Pacific, Indian and Atlantic Oceans. The variability amplitudes show strong regional differences, consistent with satellite observations (Figs. 1, 3, 6, 7, 11, 12, 14 and 15). While at least more than 45% of the global mean SLR over the past century has been attributed to anthropogenic climate change, interannual and decadal variability of coastal sea level mainly reflects internal climate variability, with a large fraction being associated with climate modes.

Over the Pacific Ocean, ENSO, PDO/IPO and NPGO are the dominant climate variability modes, which exert significant influence on coastal sea level (Sect. 3). Variations of trade wind in the equatorial Pacific related to ENSO (IPO) drive eastward-propagating equatorial Kelvin waves and westward-propagating Rossby waves, causing an east–west SLA seesaw that dominates coastal SLAs at interannual (decadal) timescales along the eastern and western boundaries of the tropical basin. Upon impinging on the eastern boundary, part of the equatorial Kelvin waves’ energy propagates poleward as coastally trapped waves, resulting in coherent SLAs all the way through Alaska in the North and southern tip of South America in the South (Figs. 2 and 3; Sect. 3.2). North of San Francisco, the effects of longshore wind and sea level pressure (atmospheric loading or IB effect), with a large part being associated with the PDO, increase with latitudes on interannual timescales, but remote forcing from the tropics remains the dominant factor for decadal SLAs. The PDO and NPGO indices, however, are least effective in capturing sea level variance along the coasts of Northeast Pacific, whereas the NOI, NPI and PNA, which are significantly correlated with the PDO and IPO, are much more effective predictors there (Fig. 4). This is likely because NOI, NPI and PNA can better capture the local longshore wind and air pressure than the PDO and NPGO indices. In extratropical western North and South Pacific, westward-propagating Rossby waves driven by interior wind stress curl related to ENSO, PDO/IPO and NPGO are the dominant force for interannual and decadal SLAs around the coasts and marginal seas. Rossby waves can excite coastally trapped waves upon impinging on the coasts and affect the location and transport of the western boundary currents and thus coastal SLAs (Sect. 2; Fig. 5). Local longshore wind and wind stress curl within the marginal seas can also be important in some coastal regions (Sect. 3.3).

Over the Indian Ocean, the most influential climate modes are ENSO, IOD and SIOD plus monsoon variability. Large-amplitude interannual and decadal SLAs occur along the eastern boundary and over the Seychelles–Chagos islands (SCTR) region (Sect. 4.1; Figs. 7c, 8 and 9). Along the Australian west coast, remote forcing by trade wind in the equatorial Pacific related to ENSO (IPO) via the ITF is the dominant cause for interannual (decadal) SLAs for all seasons, while IPO-associated wind over the Indian Ocean also plays a comparable role for decadal SLAs. From Java to the northern Bay of Bengal, coastal SLAs are primarily forced by equatorial winds at both interannual and decadal timescales, and IOD likely dominates ENSO for interannual SLAs; but quantitative assessment on their relative importance remains to be done. Along the east coast of India and Sri Lanka, longshore wind and wind stress curl in the Bay of Bengal interior are equally important as equatorial wind forcing in driving interannual SLAs during boreal summer. On decadal timescales, IPO dominates coastal SLAs during northern winter, but IOD and monsoon dominate during summer (Fig. 9). Over the SCTR region, Indian Ocean winds associated with ENSO (IPO) are the major cause for interannual (decadal) SLAs, with IOD and monsoon also having significant contributions. The SIOD has its largest influence on SLAs over the SCTR and Mascarene island regions, with negligible effect on the eastern and north boundaries of the Indian Ocean. Along the western boundary, monsoon variability appears to have larger impact on decadal SLAs compared to decadal ENSO (or IPO) and IOD (Fig. 9), and SIOD is associated with weak SLAs along the Somali coast (Fig. 10). Although Rossby waves associated with ENSO and IOD have been discussed, their impacts on coastal SLAs have not been explicitly examined. Even though the multidecadal trend of Indian Ocean sea level (with global SLR removed) since the 1960s was attributed largely to internal variability, contributions by climate modes remain unclear.

In the Atlantic Ocean, the dominant climate modes are NAO and AMO. Along the east coast of the South Atlantic, coherent interannual SLAs were observed and attributed to remote forcing from the equator. Along the east coast of the North Atlantic, Ekman heat transport associated with variations of subtropical gyre is the major cause for SLAs south of ~ 27°N. North of ~ 27°N particularly along the European coast, local and remote longshore wind (integrated from the equator), which drive coastally trapped waves, together with air pressure associated with the NAO are the major causes for interannual SLAs (Sect. 5.2). On decadal timescales, the effects of NAO may dominate coastal SLAs during some decades and AMO dominate during other decades (Figs. 13a and 15). Along the west coast of the North Atlantic, instraseasonal and interannual SLAs along the US northeast coast north of Hatteras results mainly from longshore wind forcing and IB effect; the influence of NAO is strong during some decades but weak during others, depending on its influence on longshore wind. No correlation was found between intraseasonal and interannual SLAs along New England coast and AMOC geostrophic transport, based on tide gauge and RAPID observations from 2004 to 2017. On decadal timescales, modeling experiments suggest that SLAs along US northeast coast are linked to AMOC changes, which are largely driven by basin-wide buoyancy flux associated with the NAO over the North Atlantic, with ~ 1.5 cm SLR corresponding to ~ 1 Sv AMOC weakening. Observational evidence, however, has not been established due to the short data records. South of Cape Hatteras and in subpolar region, westward-propagating Rossby waves and subsequently coastally trapped waves are the major causes for interannual and decadal SLAs. Rossby waves can affect the location and transport of the Gulf Stream and thus coastal SLAs (Sect. 5.3). Along the west coast of South Atlantic, ENSO-associated wind, sea level pressure and precipitation near the coast were suggested to be the major cause for coastal SLAs. The impacts of Rossby waves and AMOC on coastal SLAs remain elusive.

### Science Issues and Future Outlook

*(a) Coastal and open*-*ocean connections*. Currently, our understanding of the open-ocean impact on coastal sea level is based primarily on linear ocean models using idealized continental shelf and slope geometry. In the real ocean, however, shelf, slope and bottom shape vary largely from region to region. To what extent the remote equatorial SLA signals are trapped to the eastern boundary of each ocean basin as coastally trapped waves, and to what extent the open-ocean SLAs can pass through the slope barrier to arrive at the western boundary in different regions of each ocean are not well understood. From observational perspective, continued and new tide gauge records, together with satellite observations such as the Surface Water and Ocean Topography (SWOT) mission, which aims to detect sea level within 10 km from the coasts, will significantly improve our understanding of coastal and open-ocean connections. From modeling perspective, high-resolution models that can adequately resolve continental shelf, slope and western boundary currents are needed, to understand the crosstalk between coastal and the open ocean and to quantify the relative effects of coastal versus open-ocean processes on coastal sea level.

*(b) Representation of climate modes*. Climate modes are represented by their indices, which are either the principle component (PC) of an EOF mode or time series of SSTA over specific regions. Regression analyses onto these indices are used to extract their associated winds and sea level pressure that force coastal sea level variability. These methods, however, may not well represent the climate modes’ effects on sea level, because climate modes, such as ENSO dynamics, are best represented by a few eigenmodes with different periods and decay rates but nonorthogonal spatial patterns (e.g., Penland and Sardeshmukh 1995; Alexander et al. 2008; Compo and Sardeshmukh 2010; Solomon and Newman 2012). Using a fixed EOF pattern to represent climate mode (e.g., PDO) may not depict the temporal changes of their spatial patterns. Improved representations of climate modes need to be sought, in order to achieve more accurate depictions of their impacts on coastal sea level. Since local longshore wind and IB effect are important for driving coastal SLAs in extratropical oceans, it is important to make the distinction between extratropical climate indices (e.g., PDO) and tropical-centered indices (e.g., IPO), even though they are highly correlated. For instance, the PDO index can capture the PDO phase transitions in the North Pacific, whereas the IPO index cannot always capture them (Thompson et al. 2014; Merrifield and Thompson 2018). This is because the PDO is an empirical mode and not a physical mode, which reflects a superposition of multiple processes including teleconnection to the tropics, variability of Aleutian Low due to stochastic weather noise, and oceanic processes in the North Pacific (Schneider and Cornuelle 2005; Newman et al. 2016). Therefore, PDO phase transitions caused by extratropical processes may not correspond to phase changes of IPO or ENSO in the tropics.

Climate mode indices can be significantly correlated especially on decadal time scales (Sect. 3.2.2), either because of the relatively short data record that cannot make statistically significant distinctions or because they are physically connected. Robust relationships between coastal SLAs and decadal climate modes require long data records. While changes of AMOC and Gulf Stream were thought to be important for causing decadal SLAs along the US east coast, studying their long-term relationship using observations is challenging, because continuous observations of the Florida Current transport started only in 1982 and the RAPID observations of AMOC in 2004. Therefore, the impacts of AMOC on coastal SLAs are based exclusively on modeling studies. Long observational records are needed to confirm the modeling results. Furthermore, variations in SST indices of decadal climate modes (e.g., PDO and AMO) can be partly caused by external forcing (e.g., Booth et al. 2012; Dong et al. 2014). Due to these complications, extracting the effect of internal climate modes on coastal sea level from that of external forcing using observational analysis remains a challenge. To this end, the recently available large ensemble experiments of climate models with long integrations using anthropogenic, natural, and all forcing, respectively, will be helpful; however, climate models suffer from significant biases in simulating climate modes (e.g., Lyu et al. 2016).

*(c) Relation with global SLR*. Due to the global SLR, US east coast communities see acceleration in flooding incidents and severity; even small temporal elevated sea level due to internal variability, which were undetected in the past, can now pass a threshold and cause unpredictable flooding (Ezer and Atkinson 2014; Sweet and Park 2014; Park and Sweet 2015). When sea level increases induced by climate modes are added to global SLR and land subsidence, which is especially large in the mid-Atlantic Bight region (Boon 2012; Karegar et al. 2017), they can cause unexpected year-to-year variations in coastal sea level and flooding. For instance, El Niño years are found to be more prone for flooding; the extremely strong negative NAO was associated with higher coastal sea level along US east coast in 2009–2010, and flooding increased in many locations (2009 was one of the most flooded years in Norfolk; Ezer 2015; Goddard et al. 2015). Further research is needed to understand climate modes impacts on coastal floods in a changing climate.
